# Novel Oleanolic Acid-Tryptamine and -Fluorotryptamine Amides: From Adaptogens to Agents Targeting In Vitro Cell Apoptosis

**DOI:** 10.3390/plants10102082

**Published:** 2021-09-30

**Authors:** Uladzimir Bildziukevich, Marie Kvasnicová, David Šaman, Lucie Rárová, Zdeněk Wimmer

**Affiliations:** 1 Isotope Laboratory, Institute of Experimental Botany of the Czech Academy of Sciences, Vídeňská 1083, CZ-14220 Prague, Czech Republic; vmagius@gmail.com; 2 Department of Chemistry of Natural Compounds, University of Chemistry and Technology in Prague, Technická 5, CZ-16628 Prague, Czech Republic; 3 Department of Experimental Biology, Faculty of Science, Palacký University, Šlechtitelů 27, CZ-78371 Olomouc, Czech Republic; kvasnicova@ueb.cas.cz; 4 Laboratory of Growth Regulators, Institute of Experimental Botany of the Czech Academy of Sciences, Faculty of Science, Palacký University, Šlechtitelů 27, CZ-78371 Olomouc, Czech Republic; 5 Institute of Organic Chemistry and Biochemistry of the Czech Academy of Sciences, Flemingovo náměstí 2, CZ-16610 Prague, Czech Republic; nmrsaman@gmail.com

**Keywords:** oleanolic acid, tryptamine, fluorotryptamine, adaptogen, psychotropic drug, cytotoxicity, apoptosis, caspase

## Abstract

Background: Oleanolic acid is a natural plant adaptogen, and tryptamine is a natural psychoactive drug. To compare their effects of with the effect of their derivatives, tryptamine and fluorotryptamine amides of oleanolic acid were designed and synthesized. Methods: The target amides were investigated for their pharmacological effect, and basic supramolecular self-assembly characteristics. Four human cancer cell lines were involved in the screening tests performed by standard methods. Results: The ability to display cytotoxicity and to cause selective cell apoptosis in human cervical carcinoma and in human malignant melanoma was seen with the three most active compounds of the prepared series of compounds. Tryptamine amide of (3β)-3-(acetyloxy)olean-12-en-28-oic acid (3a) exhibited cytotoxicity in HeLa cancer cell lines (IC_50_ = 8.7 ± 0.4 µM) and in G-361 cancer cell lines (IC_50_ = 9.0 ± 0.4 µM). Fluorotryptamine amides of (3β)-3-(acetyloxy)olean-12-en-28-oic acid (compounds 3b and 3c) showed cytotoxicity in the HeLa cancer cell line (IC_50_ = 6.7 ± 0.4 µM and 12.2 ± 4.7 µM, respectively). The fluorotryptamine amide of oleanolic acid (compound 4c) displayed cytotoxicity in the MCF7 cancer cell line (IC_50_ = 13.5 ± 3.3 µM). Based on the preliminary UV spectra measured in methanol/water mixtures, the compounds 3a–3c were also found to self-assemble into supramolecular systems. *Conclusions*: An effect of the fluorine atom present in the molecules on self-assembly was observed with 3b. Enhanced cytotoxicity has been achieved in 3a–4c in comparison with the effect of the parent oleanolic acid (1) and tryptamine. The compounds 3a–3c showed a strong induction of apoptosis in HeLa and G-361 cells after 24 h.

## 1. Introduction

**Scheme 1:**
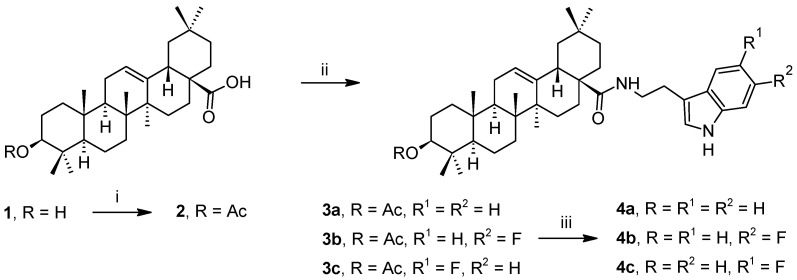
Synthetic procedure. *Reagents and conditions*: (i) acetic anhydride, DMAP, DIPEA, THF, stirring under Ar atmosphere at 90 °C, 3 h; (ii) oxalyl chloride, DCM, stirring at r.t., 3 h, tryptamine or fluorotryptamine, DIPEA, DCM:DMF (1:1), stirring at r.t., 12 h; (iii) LiOH · H_2_O, methanol, stirring at 80 °C, 4 h.

Oleanolic acid ((3β)-3-hydroxyolean-12-en-28-oic acid (1), Scheme 1) is a pentacyclic triterpenoid acid bearing a 6-6-6-6-6 skeleton of the oleanane type. It is widely distributed in the plant kingdom, often found in a form of a saponin bearing one to several monosaccharide units in the molecule [1]. Although already isolated from several hundred plant species [2,3], however, it is most often isolated from olive tree (*Olea europaea*) [1]. Oleanolic acid (1), despite its low bioavailability, also displays a broad spectrum of pharmacological activity, and it is considered to be an adaptogen [4]. It has a protective effect on the human liver, where it acts against serious liver injuries and against hepatitis [1], and it displays antitumor activity [5]. It is capable of depressing nuclear factor κB (NF-κB) [6]. Its inhibition of prostate cancer cells that normally display NF-κB activation was observed. A similar effect of triterpenoids on NF-κB was observed in relation to inflammation [6]. Oleanolic acid (1) was found to exhibit pro-apoptotic activity, and modulation of the Bcl-2 protein family due to suppression of NF-κB was found in melanoma cells. Induction of apoptosis was accompanied by activation of p53 and caspase-3 gene expression. Oleanolic acid (1) further displayed apoptosis induction in leukemia cells (HL60) through activation of caspase-9 and caspase-3, accompanied by the cleavage of poly(ADP-ribose) polymerase (PARP), as reviewed recently [6].

Cancer is one of the most widespread diseases causing the death of an important part of the human population. An intensive search for efficient drugs exhibiting selective toxicity to cancer cells and low toxicity to normal cells is urgently required. Natural products have been a potent source of novel structures for drug development [7].

Nowadays, supramolecular systems and gels and engineered nanoparticles formed in situ have emerged as advanced and promising drug delivery systems [8,9]. These types of supramolecular systems have been given increasing importance due to a possibility to manage not only drug delivery but also its pharmacological activity as a response to dynamic changes in the formed supramolecular systems [10].

Tryptamine is the most important member of the indole amine family. The tryptamine scaffold is regarded as a structure of priority importance due to its broad applications in the design of medicinal agents [11]. Tryptamine analogues have been reported to display pharmacological activity, such as anti-migraine [12], antibacterial [13,14] and antitumor effects [15,16,17]. Tryptamine itself has been found not to reduce cell viability [16]. In the area of psychotropic drugs, tryptamines have been known as a broad class of classical or serotonergic hallucinogens. The drugs are capable of producing profound changes in sensory perception, mood and thought in humans, and they act primarily as agonists of the 5-HT_2A_ receptor, a serotonin receptor family member. A tryptamine derivative psilocybin from Aztec sacred mushrooms, and another derivative, *N*,*N*-dimethyltryptamine, from South American psychoactive beverage ayahuasca, have been restrictedly used since ancient times in sociocultural and ritual contexts [18].

Melatonin (*N*-acetyl-5-methoxytryptamine) is mainly secreted by the pineal gland in brain at night, and it plays the role in regulating sleep patterns. It displays antioxidant, anti-inflammatory, and oncostatic activity. Based on recent studies, melatonin exhibits antitumor properties on different cancer types, and it may suppress cancer development in vitro and in animal models [19,20].

Fluorine can change the properties of biologically active compounds and can influence the metabolism and distribution of drug molecules in the body. Substitution by fluorine atoms at various positions of the aromatic ring of the neurotransmitter norepinephrine produced large differences in biological activity. Fluorinated melatonin analogues have enhanced activity at the pituitary melatonin receptor and increased biological half-life [21]. Fluorine is a small atom (van der Waals atomic radius is 147 pm), often mimicking hydrogen (van der Waals atomic radius 120 pm) in analogs of natural products, but it possesses higher electronegativity than hydrogen atom [22]. Due to its van der Waals atomic radius, fluorine atom has smaller atomic volume than methyl, hydroxyl or amino group [23]. Its introducing into a molecule often results in substantial modifications of the pharmacological effect of the target molecule [24]. Application of fluorine atom or trifluoromethyl group as structural modifiers of biologically active compounds has appeared since 1970s [24]. One of the pioneering compounds bearing a fluorine atom in its molecule was (9α)-9-fluorohydrocortisone, studied for its effect on blood pressure in patients with diabetes [25]. At present, the importance of fluorine-bearing compounds has been much higher in treating cancer [26] or inflammation, and it has also appeared in medicaments against depression and supporting the central nervous system [18,21,27].

Recently, several review papers have been published in pentacyclic triterpenoids and their structural modifications, including those resulting in the investigation of novel nitrogen-containing compounds [28,29,30,31,32]. However, neither of those review articles [28,29,30,31,32], nor any of the recent research papers [33,34,35] have dealt with tryptamine or fluorotryptamine derivatives of oleanolic acid that are the topic of this article as newly designed compounds.

The objectives of this investigation were: (a) to develop a synthesis of tryptamine and fluorotryptamine amides of oleanolic acid; (b) an investigation of their pharmacological effect with focusing on in vitro cytotoxicity and antiproliferative activity (cell cycle); (c) targeting this investigation on the effect of the novel compounds on the ability to cause cell apoptosis in cancer cells and to increase the caspase-3/7 activity; (d) an introductory investigation of supramolecular self-assembly of the target compound, and (e) comparing the in silico calculations of physico-chemical and ADME parameters of the target compounds with the experimentally obtained results, and finding if the novel compounds may still have a potential to display the effects of their parent compounds.

## 2. Results and Discussion

### 2.1. Designing the Synthesis and Synthetic Protocol

To begin designing the target compounds, we took into account the known low solubility of 1 in water, and, therefore, its low bioavailability. Designing amides of 1 with tryptamine derivatives (compounds 3a–4c; Scheme 1) was expected to result in compounds bearing a secondary amino group in the molecule capable of improving this characteristic in the target compounds. Moreover, as mentioned above, tryptamines display a broad spectrum of activities, including psychotropic effects. As stated above, oleanolic acid (1), despite its low bioavailability, also displays a broad spectrum of pharmacological activity. The intention of this investigation consisted in targeting new compounds with pharmacological effects enhancing and/or changing the effects of the parent compounds, and representing new cytotoxic drugs capable of causing cell apoptosis and caspase activation by combining the pharmacological characteristics of oleanolic acid (1) and tryptamine derivatives.

In the practical synthetic protocol, recently modified synthetic procedures [36] were applied to enable easy amide bond formation in the C(17)-COOH group. In the first step (Scheme 1), the C(3)-OH group in oleanolic acid (1) was converted into its acetate 2, accessible by the reaction of 1 with acetic anhydride in THF in the presence of DIPEA and DMAP as reaction catalysts in a 94% yield. Subsequently, the carboxyl group in 2 was converted into its acyl chloride using oxalyl chloride as a reagent, and the crude product was directly used to form a new amide bond in 3a–3c with tryptamine or fluorotryptamines in DCM/DMF (1:1, *v*/*v*), using DIPEA as a reaction enhancer. The products 3a–3c were obtained in about 90% yields after usual work up and chromatographic purification. Finally, the protecting acetyl group was removed by LiOH in methanol. Purification of the crude products by column chromatography gave compounds 4a–4c in >90% yields.

### 2.2. Cytotoxicity

The compounds 1, 2 and 3a–4c were tested for cytotoxicity in vitro towards the human cancer cell lines CEM (T-lymphoblastic leukemia), MCF7 (breast carcinoma), HeLa (cervical carcinoma) and G-361 (malignant melanoma). Human fibroblasts (BJ) were used as the reference cells, and CDDP [*cis*-diamminedichloridoplatinum(II), cisplatin], a pharmacologically used agent for a treatment of number of cancers, was used as a positive reference compound (Table 1). All tested compounds showed no toxicity towards the reference BJ and leukemia CEM cells (IC_50_ values were higher than 50 µM), but they showed remarkable selectivity towards the HeLa cancer cell line after 72 h. The compounds 3a and 3b showed comparable cytotoxicity towards HeLa cell line, while the cytotoxicity of 3c was lower. Based on this result, the effect of the fluorine atom in the molecule was not unequivocally established. It can be hypothesized that the position of the fluorine atom has an effect on the cytotoxicity of 3b in the HeLa cancer cell line (cf. cytotoxicity of 3b and 3c). The compounds 4b and 4c showed only low cytotoxicity in the HeLa cell line, while 4a was inactive towards all tested cell lines. In addition, 3a and 3b were cytotoxic towards the G-361 cancer cell line, and 4c showed cytotoxicity towards the MCF7 cell line, which also indicates a presumptive positive effect of the fluorine substituent on cytotoxicity of 4c. The compounds 3a and 3b showed better cytotoxicity characteristics in HeLa and G-361 cancer cell lines than CDDP, used as a reference compound (Table 1), while 3a–3c showed no toxicity in the BJ cells. Therapeutic index values were calculated for the compounds 3a–3c and 4b–4c in HeLa, G-361 and MCF7 cancer cells (Table 1). Some more details are presented in the Supplementary material, and in Table S1 therein.

### 2.3. Cell Cycle and Apoptosis

**Figure 1:**
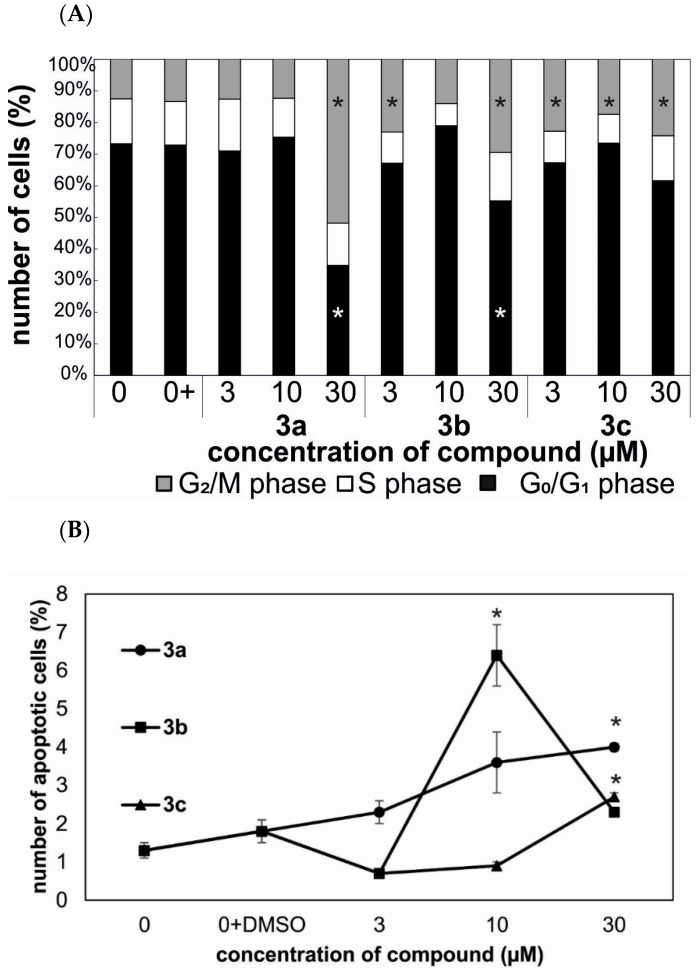
Flow cytometric analysis of cell cycle of HeLa cancer cells (**A**) and apoptosis (**B**). Flow cytometric analysis of cell cycle (**A**) and apoptosis (**B**) of human cervical carcinoma cells (HeLa) after 24 h with 3a–3c (3, 10 and 30 µM). The bars 0 means control untreated cells, the bars 0+ means control with DMSO. The experiment was repeated at least twice in triplicates. All data were analyzed by statistical software. Significance is indicated with an asterisk (*) (*p* ˂ 0.05).

The most active compounds 3a–3c were chosen from the cytotoxicity screening of a series of six prepared target compounds. All additional experiments (Western blotting, caspase activity and flow cytometry) were performed with HeLa and G-361 cells after 24 h of treatment with 3a–3c in concentrations *c* = 3, 10 or 30 µM. In cervical carcinoma (HeLa) cells, the compound 3a (*c* = 30 µM) caused cell cycle arrest in the G_2_/M phase. In addition, compounds 3b and 3c (*c* = 30 µM) increased the number of cells in the G_2_/M phase with a concomitant decrease of cells in the G_0_/G_1_ phase (Figure 1A). The strongest induction of apoptosis (up to 6.4%) was observed in HeLa cells treated by 3b (*c* = 10 µM) for 24 h. Compounds 3a (4% at *c* = 30 µM) and 3c (2.7% at *c* = 30 µM) increased the number of apoptotic cells in a dose-dependent manner, however, the effect was weaker than with 3b (Figure 1B). In contrast, the effect of 3a–3c was weaker in malignant melanoma cells (G-361) after 24 h than in cervical carcinoma cells (HeLa). G-361 cells treated with either 3a or 3b (*c* = 10 µM) or with 3c (*c* = 3 µM) for 24 h showed an increase of accumulation of the S-phase cells with concomitant decrease of the G_2_/M phase cells. G_0_/G_1_ phase of G-361 cells remained unchanged after the same treatment (Figure S1A). Compounds 3a and 3b increased the level of apoptotic cells in subG_1_ phase of melanoma cells up to 7.7% for 3a (Figure S1B).

**Figure 2:**
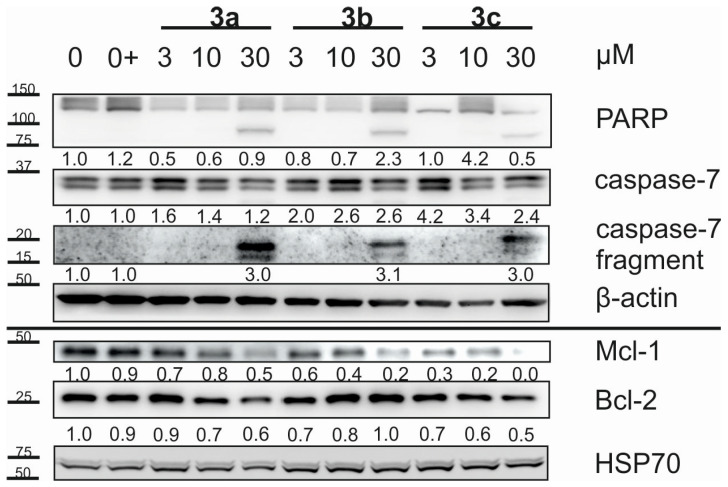
The level of protein markers of apoptosis. The level of protein markers of apoptosis (PARP, caspase-7 zymogen and fragment, Mcl-1, Bcl-2) detected by Western blotting. β-Actin or HSP70 was used as loading control. HeLa cells were treated for 24 h with 3a–3c (3, 10 and 30 µM). The bars 0 are control untreated cells, the bars 0+ are control cells with DMSO. The results presented here are based on the representative experiment repeated three times. All data were analyzed by statistical software. Signal intensities of bands were quantified by MultiGauge software (FujiFilm) and normalized to loading (β-actin or HSP70). The quantification is presented as the number below each band (x-times).

The incidence of apoptosis by 3a–3c after 24 h in HeLa cells measured by flow cytometry is in a good correlation with the results from the Western blotting, in which the cell apoptosis was confirmed. Both cell lines (HeLa and G-361) showed a strong induction of apoptosis after 24 h of treatment with all three substances when analyzed by Western blotting. The most active compounds were 3a and 3b, while 3c displayed lower activity than 3a and 3b. Since all tested compounds induced the apoptosis in HeLa and G-361 cells, the typical hallmarks of initiation of apoptosis occurred in the treatments of cells with 3a–3c at a concentration *c* = 30 µM only: a fragment of the executioner caspase-7, cleavage of its substrate, poly(ADP-ribose) polymerase (PARP), decrease of anti-apoptotic protein Mcl-1 and Bcl-2 (Figure 2; Figure S2 and Graph S in Supplementary Material).

**Figure 3:**
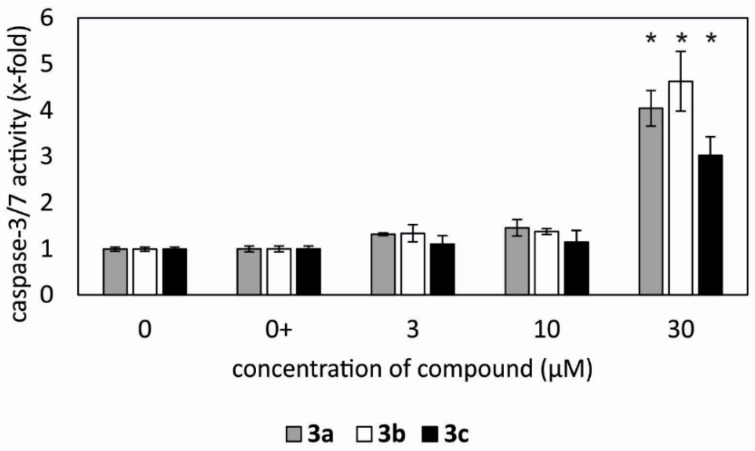
Activity of caspase-3/7. Activity of caspase-3/7 after 24 h treatment with 3a–3c (3, 10 and 30 µM) in HeLa cells. The bars 0 are control untreated cells, the bars 0+ are control cells with DMSO. The experiment was repeated at least twice in duplicates. All data were analyzed by statistical software. Significance is indicated with an asterisk (*) (*p* ˂ 0.05).

According to the Western blotting results, the activity of caspase-3/7 in HeLa and G-361 cells was correlated for the same time point and doses of 3a–3c. In HeLa cells, the activity of caspase-3/7 was induced in a dose-dependent manner for all tested compounds. The strongest induction was detected with 3a (*c* = 30 µM; up to 4 times) and 3b (*c* = 30 µM; up to 4.6 times) in HeLa cells after 24 h (Figure 3). The level of caspase-3/7 activity was lower in G-361 cells with 3a and 3b (Figure S3). In summary, compounds 3a–3c showed a strong induction of apoptosis in cervical carcinoma and malignant melanoma cells after 24 h.

### 2.4. In Silico Calculated Physico-Chemical and ADME Parameters

The experimental data determined for the compounds 1–4c were compared with the physico-chemical and ADME parameters calculated in silico using the ACD/iLabs software and databases [37]. The calculated physico-chemical parameters were further compared with the Lipinski rule of five [38] and with the Ghose rule [39]. The rules describe molecular properties important for small molecule drug pharmacokinetics in vitro, including their absorption, distribution, metabolism and excretion (known as ADME parameters). However, the rules do not predict whether pharmacological activity will be displayed. We described those rules and their importance in designing pharmacologically prospective structures recently [36]. Based on the recommended ranges given for several parameters (Table 2) it is evident that only 1 and 2 are consistent with most of those recommended ranges of the parameters, namely log *P*, log *S*, number of donor, acceptor and movable bonds, and probability of bioavailability. In turn, 1 and 2 displayed no cytotoxicity towards any of the tested cancer cell lines, while a majority of the compounds of the series of 3a–4c did show cytotoxicity (Table 1), and 3a–3c inhibited caspase-3/7 and caused cell apoptosis (Figure 2; Figure S2). Based on the values calculated for the log *PS* and log PS*f_u, brain_ parameters (Table 2) it is evident that neither of the new compounds (3a–4c) can be considered as an adaptogen. Therefore, the novel amides originated from oleanolic acid (1) and tryptamines do not display the effects of their parent compounds, because they display cytotoxicity and cause cell apoptosis (cf. Figure S4 and the accompanying text in Supplementary Material).

### 2.5. Investigation of Supramolecular Self-Assembly by UV Spectrometry

UV spectroscopy represents a simple tool to detect possible self-assembly of the target compounds, and spectra were measured in methanol/water systems containing identical concentrations of the studied compound in solutions with changing ratios of methanol/water mixture (in 20% concentration steps). With no self-assembly occurring in the investigated system, the absorbance decreased regularly with a decreasing concentration of methanol in the methanol/water mixtures. However, when self-assembly occurred in the solutions of the studied compounds during the UV measurements within the time interval of 96 h, irregularity of absorbance value either with a decreasing concentration of methanol in the methanol/water mixture or with an increasing time of the measurement appears in the respective sequence of concentrations or time intervals. A more detailed information is presented in Figure S5 (Supplementary Material).

This basic investigation of supramolecular self-assembly was done because we had found a relationship between cytotoxicity and ability of compounds to form supramolecular aggregates capable of affecting cytotoxicity [10,35]. Similar results have recently been published by other authors [40,41]. More detailed information is presented in Table S1 (Supplementary Material).

## 3. Materials and Methods

### 3.1. General

The NMR measurements were performed either on an AVANCE II 600 MHz spectrometer (Bruker, Berlin, Germany) equipped with a 5 mm TCI cryoprobe or on a Bruker AVANCE III 500 MHz spectrometer in a 5 mm tube in different solvents. The ^1^H and ^13^C NMR spectra were recorded at 600.13 MHz or 150.90 MHz, respectively, on a 600 MHz instrument, and the ^19^F-NMR spectra were recorded at 470.40 MHz on a 500 MHz instrument, in CDCl_3_ or CD_3_OD using tetramethylsilane (δ = 0.0 − CDCl_3_) or signal of the solvent (δ = 3.31 or 49.50 for ^1^H/^13^C − CD_3_OD) as internal references. ^1^H NMR data are presented in the following order: chemical shift (δ) expressed in ppm, multiplicity (s, singlet; d, doublet; t, triplet; q, quartet; m, multiplet), number of protons, coupling constants in Hertz. For unambiguous assignment of both ^1^H and ^13^C signals, 2D NMR, ^1^H, ^13^C gHSQC and gHMBC spectra, were measured using standard parameters sets and pulse programs delivered by producer of the spectrometer. A Nicolet iS5 FT-IR spectrometer (ThermoFisher Scientific, Waltham, MA, USA) was used for recording IR spectra. Mass spectra (MS) were measured with a ZMD mass spectrometer (Waters, Eschborn, Germany) in a positive ESI mode (coin voltage, CV = 10 to 20 V). A Perkin Elmer 2400 Series II CHNS/O Analyzer (Perkin Elmer, Richmond, CA, USA) was used for simultaneous determination of C, H and N (accuracy of CHN determination better than 0.30% abs.). TLC was carried out on silica gel plates (Merck 60F_254_) and the visualization was performed by both, the UV detection and spraying with the methanolic solution of phosphomolybdic acid (5%) followed by heating. For column chromatography, silica gel 60 (0.063–0.200 mm) from Merck (Darmstadt, Germany) was used. Analytical HPLC was carried out on a TSP (Thermoseparation Products, Waltham, MA USA) instrument equipped with a ConstaMetric 4100 Bio pump and a SpectroMonitor 5000 UV DAD. The analyses of the products were performed on a reverse phase Nucleosil 120-5 C18 column (250 × 4 mm; Watrex, Prague, Czech Republic) using a methanol/water mixture (9:1, *v*/*v*) as mobile phase at 0.5 to 1.0 mL.min^−1^. The eluate was monitored at 220, 254, and 275 nm, and the UV spectra were run from 200 to 300 nm. All chemicals and solvents were purchased from regular commercial sources in analytical grade and the solvents were purified by general methods before use. Oleanolic acid was purchased from Dr. Jan Šarek – Betulinines (Stříbrná Skalice, Czech Republic; www.betulinines.com, (accessed on 23 September 2021).

### 3.2. Synthesis of Compound *2*

Acetic anhydride (33 mg), DMAP (35 mg) and DIPEA (1 mL) were added to a solution of oleanolic acid (1, 1000 mg) in THF (10 mL). The reaction mixture was heated under stirring in an argon atmosphere at 90 °C for 3 h. The reaction was then stopped by adding water, and volatile components were removed under reduced pressure. The mixture was extracted with chloroform (3 × 20 mL), the extract was dried with sodium sulfate, and then the solvent was evaporated under reduced pressure. Column chromatography afforded 2 in a 94% yield as colorless oil. ^1^H-NMR: δ 0.73 (3H, s, H26), 0.79 (1H, dd, *J*_1_ = 1.8 Hz, *J*_2_ = 11.5 Hz, *J*_3_ = 13.2 Hz, H5), 0.80 (3H, s, H25), 0.82 (3H, s, H23), 0.85 (3H, s, H24), 0.88 (3H, s, H29), 0.88 (3H, d, *J* = 0.6 Hz, H30), 1.03 (2H, ddd, *J*_1_ = 3.0 Hz, *J*_2_ = 3.9 Hz, *J*_3_ = 14.0 Hz, H16), 1.09 (3H, d, *J* = 0.8 Hz, H27), 1.70 (2H, dt, *J*_1_ = 4.4 Hz, *J*_2_ = 13.9 Hz, *J*_3_ = 13.9 Hz, H11), 1.81 (2H, ddd, *J*_1_ = 3.3 Hz, *J*_2_ = 7.2 Hz, *J*_3_ = 18.4 Hz, H2), 1.85 (2H, ddd, *J*_1_ = 3.2 Hz, *J*_2_ = 10.0 Hz, *J*_3_ = 18.4 Hz, H2), 1.92 (2H, dt, *J*_1_ = 4.1 Hz, *J*_2_ = 13.6 Hz, *J*_3_ = 13.6 Hz, H16), 2.00 (3H, s, H2′), 2.78 (1H, ddd, *J*_1_ = 4.6 Hz, *J*_2_ = 13.8 Hz, H18), 4.44 (1H, dd, *J*_1_ = 3.2 Hz, *J*_2_ = 10.0 Hz, H3), 5.23 (1H, t, *J* = 3.8 Hz, H12). ^13^C- NMR: δ 15.24 (q, C24), 16.53 (q, C25), 16.73 (q, C26), 18.10 (t, C6), 21.17 (q, C2′), 22.90 (t, C11), 23.28 (t, C2), 23.39 (t, C16), 23.44 (q, C30), 25.72 (q, C27), 27.55 (t, C15), 27.90 (q, C23), 30.57 (s, C20), 32.41 (t, C22), 32.52 (t, C7), 32.95 (q, C29), 33.75 (t, C21), 36.82 (s, C10), 37.57 (s, C4), 37.97 (t, C1), 39.14 (s, C8), 41.07 (s, C14), 41.60 (d, C18), 45.82 (s, C17), 46.29 (t, C19), 47.43 (d, C9), 55.18 (d, C5), 81.10 (d, C3), 122.11 (d, C12), 143.76 (s, C13), 171.38 (s, C1′), 180.72 (s, C28). IR (cm^−1^): 2980, 2932, 2855, 1733, 1689, 1460, 1031. MS (CV = 20 V): *m*/*z* = 516.2 [M+NH_4_]^+^. For C_32_H_50_O_4_ (498.74) calcd. C (77.06), H (10.10), found C (77.03), H (10.11).

### 3.3. Synthesis of *3a*–*3c*

Oxalyl chloride (0.4 mL) was added to a solution of 2 (51 mg) in DCM (2 mL), the mixture was stirred for 3 h, and then the excess of oxalyl chloride was evaporated. The crude acyl chloride was added to a solution of tryptamine or fluorotryptamines (24 mg) in DCM (2 mL), DMF (2 mL) and DIPEA (0.08 mL), and the mixture was stirred for 12 h. Evaporation of the solvents yielded the crude product that was purified by column chromatography, finally affording 3a–3c in 87-89% yields as white crystalline compounds.

Compound 3a: ^1^H-NMR: δ 0.40 (3H, s, H25), 0.74 (1H, dd, *J*_1_ = 1.8 Hz, *J*_2_ = 11.4 Hz, H5), 0.75 (3H, d, *J* = 0.6 Hz, H24), 0.83 (3H, s, H26), 0.83 (3H, s, H23), 0.88 (3H, s, H29), 0.89 (3H, s, H30), 1.01 (2H, ddd, *J*_1_ = 2.6 Hz, *J*_2_ = 4.6 Hz, *J*_3_ = 13.6 Hz, H19), 1.02 (3H, d, *J* = 0.7 Hz, H27), 1.50 (2H, dt, *J*_1_ = 3.5 Hz, *J*_2_ = 3.5 Hz, *J*_3_ = 13.1 Hz, H1), 1.65 (2H, t, *J*_1_ = 13.4 Hz, *J*_2_ = 13.4 Hz, H19), 1.75 (2H, ddd, *J*_1_ = 3.0 Hz, *J*_2_ = 4.1 Hz, *J*_3_ = 14.2 Hz, H22), 1.87 (2H, dt, *J*_1_ = 3.9 Hz, *J*_2_ = 13.7 Hz, *J*_3_ = 13.7 Hz, H16), 2.04 (3H, s, H13′), 2.21 (1H, ddd, *J*_1_ = 4.5 Hz, *J*_2_ = 13.3 Hz, H18), 2.88 (2H, dddd, *J*_1_ = 0.5 Hz, *J*_2_ = 6.2 Hz, *J*_3_ = 9.9 Hz, *J*_4_ = 14.3 Hz, H2′), 3.03 (2H, dddd, *J*_1_ = 1.0 Hz, *J*_2_ = 4.6 Hz, *J*_3_ = 5.5 Hz, *J*_4_ = 14.3 Hz, H2′), 3.17 (2H, dddd, *J*_1_ = 0.9 Hz, *J*_2_ = 5.5 Hz, *J*_3_ = 9.9 Hz, *J*_4_ = 13.1 Hz, H1′), 3.96 (2H, dddd, *J*_1_ = 4.6 Hz, *J*_2_ = 6.2 Hz, *J*_3_ = 8.0 Hz, *J*_4_ = 13.1 Hz, H1′), 4.40 (1H, t, *J* = 3.7 Hz, H12), 4.44 (1H, dd, *J*_1_ = 5.9 Hz, *J*_2_ = 10.5 Hz, H3), 5.98 (1H, dd, *J*_1_ = 2.9 Hz, *J*_2_ = 8.0 Hz, H1′-NH), 7.05 (1H, dd, *J* = 1.0 Hz, H4′), 7.13 (1H, ddd, *J*_1_ = 1.0 Hz, *J*_2_ = 7.0 Hz, *J*_3_ = 9.0 Hz, H9′), 7.21 (1H, ddd, *J*_1_ = 1.2 Hz, *J*_2_ = 7.0 Hz, *J*_3_ = 8.2 Hz, H8′), 7.39 (1H, ddd, *J*_1_ = 0.9 Hz, *J*_2_ = 0.9 Hz, *J*_3_ = 8.2 Hz, H7′), 7.61 (1H, dt, *J*_1_ = 1.0 Hz, *J*_2_ = 1.0 Hz, *J*_3_ = 8.0 Hz, H10′), 8.17 (1H, ds, H5′-NH). ^13^C-NMR: δ 15.33 (q, C24), 16.42 (q, C25), 16.60 (q, C26), 18.06 (t, C6), 21.30 (q, C13′), 23.18 (t, C11), 23.46 (t, C2), 23.54 (q, C30), 23.71 (t, C16), 24.79 (t, C2′), 25.61 (q, C27), 27.14 (t, C15), 27.95 (q, C23), 30.66 (s, C20), 32.04 (t, C22), 32.24 (t, C7), 32.91 (q, C29), 34.12 (t, C21), 36.67 (s, C10), 37.61 (s, C4), 38.07 (tt, C1), 39.15 (s, C8), 39.53 (t, C1′), 41.71 (s, C14), 41.90 (d, C18), 46.21 (s, C17), 46.65 (t, C19), 47.26 (d, C9), 55.04 (d, C5), 80.83 (d, C3), 111.27 (d, C7′), 113.12 (s, C3′), 118.78 (d, C10′), 119.73 (d, C9′), 122.02 (d, C4′), 122.37 (s, C11′), 122.42 (d, C8′), 122.65 (d, C12), 136.51 (s, C6′), 144.06 (s, C13), 171.02 (s, C12′), 178.27 (s, C28). IR (cm^−1^): 3265, 2980, 2932, 2855, 1733, 1689, 1621, 1460, 1109, 1031. MS (CV = 10 V): *m*/*z* = 641.4 [M+H]^+^. For C_42_H_60_N_2_O_3_ (640.94) calcd. C (78.70), H (9.44), N (4.37), found C (78.67), H (9.45), N (4.39). Melting point 130–131 °C.

Compound 3b: ^1^H-NMR: δ 0.41 (3H, s, H25), 0.74 (1H, *J*_1_ = 1.8, *J*_2_ = 11.5 Hz, H5), 0.76 (3H, s, H24), 0.83 (3H, s, H26), 0.83 (3H, s, H23), 0.88 (3H, s, H30), 0.88 (3H, s, H29), 1.04 (2H, ddd, *J*_1_ = 2.6 Hz, *J*_2_ = 4.7 Hz, *J*_3_ = 13.6 Hz, H19), 1.04 (3H, s, H27), 1.51 (2H, dt, *J*_1_ = 3.5 Hz, *J*_2_ = 3.5 Hz, *J*_3_ = 13.0 Hz, H1), 1.67 (2H, t, *J* = 13.4 Hz, H19), 1.74 (2H, ddd, *J*_1_ = 3.0 Hz, *J*_2_ = 4.0 Hz, *J*_3_ = 14.1 Hz, H22), 1.89 (2H, dt, *J*_1_ = 3.9 Hz, *J*_2_ = 13.8 Hz, *J*_3_ = 13.8 Hz, H16), 2.22 (1H, ddd, *J*_1_ = 4.2 Hz, *J*_2_ = 13.2 Hz, H18), 2.84 (2H, dddd, *J*_1_ = 6.4 Hz, *J*_2_ = 9.7 Hz, *J*_3_ = 14.5 Hz, H2′), 3.00 (2H, dddd, *J*_1_ = 1.0 Hz, *J*_2_ = 4.6 Hz, *J*_3_ = 5.6 Hz, *J*_4_ = 14.5 Hz, H2′), 3.16 (2H, dddd, *J*_1_ = 2.9 Hz, *J*_2_ = 5.6 Hz, *J*_3_ = 9.7 Hz, *J*_4_ = 12.8 Hz, H1′), 3.93 (2H, dddd, *J*_1_ = 4.6 Hz, *J*_2_ = 6.4 Hz, *J*_3_ = 8.0 Hz, *J*_4_ = 12.8 Hz, H1′), 4.45 (1H, dd, *J*_1_ = 5.9 Hz, *J*_2_ = 10.2 Hz, H3), 4.56 (1H, t, *J* = 3.6 Hz, H12), 5.96 (1H, dd, *J*_1_ = 2.9 Hz, *J*_2_ = 8.0 Hz, H1′-NH), 6.89 (1H, ddd, *J*_1_ = 2.3 Hz, *J*_2_ = 8.7 Hz, *J*_3_ = 9.5 Hz, H9′), 7.02 (1H, bd, *J* = 2.2 Hz, H4′), 7.07 (1H, dd, *J*_1_ = 2.3 Hz, *J*_2_ = 9.5 Hz, H7′), 7.50 (1H, dd, *J*_1_ = 5.2 Hz, *J*_2_ = 8.6 Hz, H10′), 8.31 (1H, bs, H5′-NH). ^13^C-NMR: δ 15.19 (q, C24), 16.44 (q, C25), 16.60 (q, C26), 18.06 (t, C6), 21.30 (q, C13′), 23.23 (t, C11), 23.45 (t, C2), 23.50 (q, C30), 23.77 (t, C16), 24.88 (t, C2′), 25.61 (q, C27), 27.13 (t, C15), 27.95 (q, C23), 30.66 (s, C20), 32.05 (t, C22), 32.27 (t, C7), 32.89 (q, C29), 34.10 (t, C21), 36.64 (s, C10), 37.61 (s, C4), 38.08 (t, C1), 39.17 (s, C8), 39.24 (t, C1′), 41.77 (s, C14), 42.02 (d, C18), 46.23 (s, C17), 46.66 (t, C19), 47.25 (d, C9), 55.04 (d, C5), 80.83 (d, C3), 97.58 (d, C7′), 108.45 (d, C9′), 113.22 (s, C3′), 119.55 (d, C10′), 122.32 (d, C4′), 122.62 (d, C12), 123.94 (s, C11′), 136.47 (s, C6′), 144.26 (s, C13), 160.19 (s, C8′), 171.04 (s, C12′), 178.25 (s, C28). ^19^F-NMR: δ 117.4 (dt, *J*_1_ = 5.2 Hz, *J*_2_ = 9.5 Hz, *J*_3_ = 9.5 Hz). IR (cm^−1^): 3260, 2981, 2931, 2855, 1734, 1690, 1621, 1461, 1110, 1031. MS (CV = 20 V): *m*/*z* = 659.3 [M+H]^+^. For C_42_H_59_FN_2_O_3_ (658.93) calcd. C (76.56), H (9.03), F (2.88), N (4.25), found C (76.53), H (9.05), F (2.90), N (4.21). Melting point 139–140 °C.

Compound 3c: ^1^H-NMR: δ 0.44 (3H, s, H25), 0.74 (3H, d, *J* = 0.6 Hz, H24), 0.74 (1H, dd, *J*_1_ = 1.9 Hz, *J*_2_ = 11.5 Hz, H5), 0.83 (3H, s, H26), 0.83 (3H, s, H23), 0.87 (3H, s, H30), 0.87 (3H, s, H29), 1.04 (2H, ddd, *J*_1_ = 2.5 Hz, *J*_2_ = 3.5 Hz, *J*_3_ = 13.6 Hz, H19), 1.04 (3H, d, *J* = 0.7 Hz, H27), 1.50 (2H, dt, *J*_1_ = 3.5 Hz, *J*_2_ = 3.5 Hz, *J*_3_ = 13.1 Hz, H1), 1.67 (2H, t, *J* = 13.3 Hz, H19), 1.74 (2H, ddd, *J*_1_ = 3.0 Hz, *J*_2_ = 4.1 Hz, *J*_3_ = 14.2 Hz, H22), 1.88 (2H, dt, *J*_1_ = 3.8 Hz, *J*_2_ = 13.6 Hz, *J*_3_ = 13.6 Hz, H16), 2.04 (3H, s, H13′), 2.22 (1H, ddd, *J*_1_ = 4.4 Hz, *J*_2_ = 13.2 Hz, H18), 2.82 (2H, dddd, *J*_1_ = 0.5 Hz, *J*_2_ = 6.4 Hz, *J*_3_ = 9.6 Hz, *J*_4_ = 14.5 Hz, H2′), 2.97 (2H, dddd, *J*_1_ = 1.0 Hz, *J*_2_ = 4.7 Hz, *J*_3_ = 5.6 Hz, *J*_4_ = 14.5 Hz, H2′), 3.16 (2H, dddd, *J*_1_ = 3.0 Hz, *J*_2_ = 5.7 Hz, *J*_3_ = 9.6 Hz, *J*_4_ = 12.8 Hz, H1′), 3.92 (2H, dddd, *J*_1_ = 4.7 Hz, *J*_2_ = 6.4 Hz, *J*_3_ = 8.0 Hz, *J*_4_ = 12.8 Hz, H1′), 4.44 (1H, dd, *J*_1_ = 6.0 Hz, *J*_2_ = 10.5 Hz, H3), 4.54 (1H, t, *J* = 3.7 Hz, H12), 5.95 (1H, dd, *J*_1_ = 3.0 Hz, *J*_2_ = 8.0 Hz, H1′-NH), 6.96 (1H, dt, *J*_1_ = 2.4 Hz, *J*_2_ = 8.9 Hz, *J*_3_ = 8.9 Hz, H8′), 7.09 (1H, d, *J* = 2.3 Hz, H4′), 7.23 (1H, dd, *J*_1_ = 2.4 Hz, *J*_2_ = 9.4 Hz, H10′), 7.31 (1H, ddd, *J*_1_ = 0.5 Hz, *J*_2_ = 4.4 Hz, *J*_3_ = 8.8 Hz, H7′), 8.33 (1H, ds, H5′-NH). ^13^C-NMR: δ 15.23 (q, C24), 16.44 (q, C25), 16.60 (q, C26), 18.06 (t, C6), 21.30 (q, C13′), 23.21 (t, C11), 23.43 (t, C2), 23.45 (q, C30), 23.76 (t, C16), 24.83 (t, C2′), 25.61 (q, C27), 27.13 (t, C15), 27.95 (q, C23), 30.63 (s, C20), 32.04 (t, C22), 32.25 (t, C7), 32.89 (q, C29), 34.10 (t, C21), 36.65 (s, C10), 37.61 (s, C4), 38.68 (t, C1), 39.16 (s, C8), 39.18 (t, C1′), 41.76 (d, C18), 42.04 (s, C14), 46.23 (t, C19), 46.68 (d, C9), 47.26 (s, C17), 55.06 (d, C5), 80.84 (d, C3), 103.67 (d, C10′), 110.77 (d, C8′), 111.93 (d, C7′), 113.23 (s, C3′), 122.60 (d, C12), 123.93 (d, C4′), 127.72 (s, C11′), 132.99 (s, C6′), 144.29 (s, C13), 157.83 (s, C9′), 171.06 (s, C12′), 178.27 (s, C28). ^19^F-NMR: δ 124.13 (dt, *J*_1_ = 4.4 Hz, *J*_2_ = 9.3 Hz, *J*_3_ = 9.3 Hz). IR (cm^−1^): 3260, 2980, 2933, 2857, 1734, 1687, 1619, 1459, 1108, 1030. MS (CV = 20 V): *m*/*z* = 659.3 [M+H]^+^. For C_42_H_59_FN_2_O_3_ (658.93) calcd. C (76.56), H (9.03), F (2.88), N (4.25), found C (76.59), H (9.01), F (2.87), N (4.27). Melting point 138–139 °C.

### 3.4. Synthesis of 4a–4c

LiOH·H_2_O (82 mg) was added to a solution of 3a, 3b or 3c (246 mg) in methanol (25 mL), and the mixture was stirred at 80 °C for 4 h. The mixture was filtered, and the inorganic salts were washed with chloroform. After evaporation of the solvents, the crude residue was purified by column chromatography on silica gel, using chloroform/methanol (300:1) as mobile phase. The products 4a–4c were obtained in 93–95% yields as white crystalline compounds.

Compound 4a: ^1^H-NMR: δ 0.41 (3H, s, H25), 0.63 (1H, dd, *J*_1_ = 1.8 Hz, *J*_2_ = 11.7 Hz, H5), 0.73 (3H, d, *J* = 0.6 Hz, H24), 0.76 (3H, s, H26), 0.87 (3H, s, H29), 0.89 (3H, s, H30), 0.96 (3H, s, H23), 1.01 (2H, ddd, *J*_1_ = 2.6 Hz, *J*_2_ = 4.6 Hz, *J*_3_ = 13.6 Hz, H19), 1.03 (3H, d, *J* = 0.8 Hz, H27), 1.65 (2H, t, *J* = 13.4 Hz, H19), 1.76 (2H, ddd, *J*_1_ = 3.0 Hz, *J*_2_ = 4.1 Hz, *J*_3_ = 14.1 Hz, H22), 1.87 (2H, dt, *J*_1_ = 4.0 Hz, *J*_2_ = 13.7 Hz, *J*_3_ = 13.7 Hz, H16), 2.21 (1H, ddd, *J*_1_ = 4.7 Hz, *J*_2_ = 13.1 Hz, H18), 2.88 (2H, dddd, *J*_1_ = 0.6 Hz, *J*_2_ = 6.2 Hz, *J*_3_ = 9.9 Hz, *J*_4_ = 14.5 Hz, H2′), 3.03 (2H, dddd, *J*_1_ = 1.0 Hz, *J*_2_ = 4.5 Hz, *J*_3_ = 5.4 Hz, *J*_4_ = 14.5 Hz, H2′), 3.16 (2H, dddd, *J*_1_ = 2.8 Hz, *J*_2_ = 5.3 Hz, *J*_3_ = 9.9 Hz, *J*_4_ = 13.2 Hz, H1′), 3.17 (1H, dd, *J*_1_ = 4.9 Hz, *J*_2_ = 11.5 Hz, H3), 3.97 (2H, dddd, *J*_1_ = 4.5 Hz, *J*_2_ = 6.2 Hz, *J*_3_ = 8.0 Hz, *J*_4_ = 13.2 Hz, H1′), 4.40 (1H, t, *J* = 3.7 Hz, H12), 5.98 (1H, dd, *J*_1_ = 2.8 Hz, *J*_2_ = 8.0 Hz, H1′-NH), 7.05 (1H, bs, H4′), 7.13 (1H, ddd, *J*_1_ = 1.0 Hz, *J*_2_ = 7.0 Hz, *J*_3_ = 8.0 Hz, H9′), 7.22 (1H, ddd, *J*_1_ = 1.2 Hz, *J*_2_ = 7.0 Hz, *J*_3_ = 8.2 Hz, H8′), 7.39 (1H, dt, *J*_1_ = 0.9 Hz, *J*_2_ = 0.9 Hz, *J*_3_ = 8.2 Hz, H7′), 7.61 (1H, dt, *J*_1_ = 1.0 Hz, *J*_2_ = 1.0 Hz, *J*_3_ = 8.0 Hz, H10′), 8.16 (1H, bs, H5′-NH). ^13^C-NMR: δ 15.29 (q, C24), 15.51 (q, C25), 16.44 (q, C26), 18.18 (t, C6), 23.18 (t, C11), 23.54 (q, C30), 23.72 (t, C16), 24.76 (t, C2′), 25.65 (q, C27), 27.09 (t, C2), 27.16 (t, C15), 28.02 (q, C23), 30.66 (s, C20), 32.10 (t, C22), 32.23 (t, C7), 32.91 (q, C29), 34.13 (t, C21), 36.78 (s, C10), 38.39 (t, C1), 38.70 (s, C4), 39.14 (s, C8), 39.60 (t, C1′), 41.72 (s, C14), 41.90 (d, C18), 46.20 (s, C17), 46.70 (t, C19), 47.33 (d, C9), 54.95 (d, C5), 78.93 (d, C3), 111.26 (d, C7′), 113.13 (s, C3′), 118.77 (d, C10′), 119.74 (d, C9′), 122.02 (d, C4′), 122.39 (s, C11′), 122.43 (d, C8′), 122.74 (d, C12), 136.51 (s, C6′), 144.05 (s, C13), 178.29 (s, C28). IR (cm^−1^): 3265, 2980, 2932, 2856, 1690, 1621, 1460, 1109, 1030. MS (CV = 15 V): *m*/*z* = 599.2 [M+H]^+^. For C_40_H_58_N_2_O_2_ (598.90) calcd. C (80.22), H (9.76), N (4.68), found C (80.25), H (9.75), N (4.66). Melting point 145–146 °C.

Compound 4b: ^1^H-NMR: δ 0.42 (3H, s, H25), 0.64 (1H, dd, *J*_1_ = 1.9 Hz, *J*_2_ = 11.8 Hz, H5), 0.74 (3H, s, H24), 0.76 (3H, s, H26), 0.87 (3H, s, H30), 0.87 (3H, s, H29), 0.96 (3H, s, H23), 1.03 (2H, ddd, *J*_1_ = 2.5 Hz, *J*_2_ = 4.4 Hz, *J*_3_ = 13.6 Hz, H19), 1.05 (3H, d, *J* = 0.5 Hz, H27), 1.67 (2H, t, *J* = 13.5 Hz, H19), 1.74 (2H, ddd, *J*_1_ = 3.0 Hz, *J*_2_ = 4.1 Hz, *J*_3_ = 14.1 Hz, H22), 1.88 (2H, dt, *J*_1_ = 3.9 Hz, *J*_2_ = 13.7 Hz, *J*_3_ = 13.7 Hz, H16), 2.22 (1H, ddd, *J*_1_ = 4.4 Hz, *J*_2_ = 12.9 Hz, H18), 2.84 (2H, ddd, *J*_1_ = 6.3 Hz, *J*_2_ = 9.8 Hz, *J*_3_ = 14.5 Hz, H2′), 3.00 (2H, dddd, *J*_1_ = 1.0 Hz, *J*_2_ = 4.7 Hz, *J*_3_ = 5.7 Hz, *J*_4_ = 14.5 Hz, H2′), 3.16 (2H, dddd, *J*_1_ = 2.9 Hz, *J*_2_ = 5.7 Hz, *J*_3_ = 9.8 Hz, *J*_4_ = 12.9 Hz, H1′), 3.18 (1H, dd, *J*_1_ = 4.8 Hz, *J*_2_ = 11.4 Hz, H3), 3.93 (2H, dddd, *J*_1_ = 4.7 Hz, *J*_2_ = 6.3 Hz, *J*_3_ = 7.9 Hz, *J*_4_ = 12.9 Hz, H1′), 4.55 (1H, t, *J* = 3.6 Hz, H12), 5.94 (1H, dd, *J*_1_ = 2.9 Hz, *J*_2_ = 7.9 Hz, H1′-NH), 6.90 (1H, ddd, *J*_1_ = 2.2 Hz, *J*_2_ = 8.7 Hz, *J*_3_ = 9.6 Hz, H9′), 7.02 (1H, bd, *J* = 2.2 Hz, H4′), 7.07 (1H, dd, *J*_1_ = 2.2 Hz, *J*_2_ = 9.6 Hz, H7′), 7.51 (1H, dd, *J*_1_ = 5.3 Hz, *J*_2_ = 8.7 Hz, H10′), 8.15 (1H, bs, H5′-NH). ^13^C-NMR: δ 15.16 (q, C24), 15.51 (q, C25), 16.46 (q, C26), 18.19 (t, C6), 23.24 (t, C11), 23.50 (q, C30), 23.78 (t, C16), 24.86 (t, C2′), 25.65 (q, C27), 27.11 (t, C2), 27.16 (t, C15), 28.03 (q, C23), 30.67 (s, C20), 32.11 (t, C22), 32.27 (t, C7), 32.91 (q, C29), 34.12 (t, C21), 36.76 (s, C10), 38.42 (t, C1), 38.70 (s, C4), 39.17 (t, C1′), 39.33 (s, C8), 41.77 (s, C14), 42.04 (d, C18), 46.20 (s, C17), 46.71 (d, C9), 47.34 (t, C19), 54.96 (d, C5), 78.93 (d, C3), 97.56 (d, C7′), 108.51 (d, C9′), 113.37 (s, C3′), 119.60 (d, C10′), 122.25 (d, C4′), 122.68 (d, C12), 124.00 (s, C11′), 136.42 (s, C6′), 144.31 (s, C13), 160.22 (s, C8′), 178.24 (s, C28). ^19^F NMR: δ 120.80 (dt, *J*_1_ = 5.3 Hz, *J*_2_ = 9.6 Hz, *J*_3_ = 9.6 Hz). IR (cm^−1^): 3257, 2980, 2931, 2856, 1690, 1621, 1461, 1109, 1030. MS (CV = 20 V): *m*/*z* = 617.3 [M+H]^+^. For C_40_H_57_FN_2_O_2_ (616.89) calcd. C (77.88), H (9.31), F (3.08), N (4.54), found C (77.90), H (9.29), F (3.07), N (4.56). Melting point 149–150 °C.

Compound 4c: ^1^H NMR: δ 0.44 (3H, s, H25), 0.64 (1H, dd, *J*_1_ = 1.8 Hz, *J*_2_ = 11.4 Hz, H5), 0.75 (3H, s, H24), 0.76 (3H, s, H26), 0.87 (3H, s, H30), 0.87 (3H, s, H29), 0.96 (3H, s, H23), 1.03 (2H, ddd, *J*_1_ = 2.5 Hz, *J*_2_ = 4.5 Hz, *J*_3_ = 13.6 Hz, H19), 1.05 (3H, s, H27), 1.67 (2H, t, *J* = 13.3 Hz, H19), 1.74 (2H, ddd, *J*_1_ = 3.0 Hz, *J*_2_ = 3.9 Hz, *J*_3_ = 14.1 Hz, H22), 1.88 (2H, dt, *J*_1_ = 3.3 Hz, *J*_2_ = 13.4 Hz, *J*_3_ = 13.4 Hz, H16), 2.22 (1H, ddd, *J*_1_ = 4.4 Hz, *J*_2_ = 13.4 Hz, H18), 2.83 (2H, ddd, *J*_1_ = 6.4 Hz, *J*_2_ = 9.8 Hz, *J*_3_ = 14.5 Hz, H2′), 2.97 (2H, ddd, *J*_1_ = 4.7 Hz, *J*_2_ = 5.7 Hz, *J*_3_ = 14.5 Hz, H2′), 3.16 (2H, dddd, *J*_1_ = 2.9 Hz, *J*_2_ = 5.7 Hz, *J*_3_ = 9.8 Hz, *J*_4_ = 12.9 Hz, H1′), 3.18 (1H, dd, *J*_1_ = 4.8 Hz, *J*_2_ = 11.4 Hz, H3), 3.92 (2H, dddd, *J*_1_ = 4.7 Hz, *J*_2_ = 6.4 Hz, *J*_3_ = 7.9 Hz, *J*_4_ = 12.9 Hz, H1′), 4.54 (1H, t, *J* = 3.7 Hz, H12), 5.96 (1H, dd, *J*_1_ = 2.9 Hz, *J*_2_ = 7.9 Hz, H1′-NH), 6.96 (1H, dt, *J*_1_ = 2.5 Hz, *J*_2_ = 8.9 Hz, *J*_3_ = 8.9 Hz, H8′), 7.09 (1H, bs, H4′), 7.23 (1H, dd, *J*_1_ = 2.5 Hz, *J*_2_ = 9.4 Hz, H10′), 7.30 (1H, dd, *J*_1_ = 4.2 Hz, *J*_2_ = 8.8 Hz, H7′), 8.35 (1H, bs, H5′-NH). ^13^C-NMR: δ 15.18 (q, C24), 15.51 (q, C25), 16.44 (q, C26), 18.18 (t, C6), 23.21 (t, C11), 23.43 (q, C30), 23.77 (t, C16), 24.80 (t, C2′), 25.65 (q, C27), 27.08 (t, C2), 27.15 (t, C15), 28.02 (q, C23), 30.62 (s, C20), 32.10 (t, C22), 32.25 (t, C7), 32.90 (q, C29), 34.11 (t, C21), 36.76 (s, C10), 38.41 (t, C1), 38.69 (s, C4), 39.16 (t, C1′), 39.29 (s, C8), 41.77 (s, C14), 42.02 (d, C18), 46.22 (s, C17), 46.72 (d, C9), 47.33 (t, C19), 54.96 (d, C5), 78.93 (d, C3), 103.65 (d, C10′), 110.77 (d, C8′), 111.93 (d, C7′), 113.22 (s, C3′), 122.70 (d, C12), 123.93 (d, C4′), 127.74 (s, C11′), 132.98 (s, C6′), 144.27 (s, C13), 157.84 (s, C9′), 178.30 (s, C28) ^19^F-NMR: δ 120.80 (dt, *J*_1_ = 4.2 Hz, *J*_2_ = 9.3 Hz, *J*_3_ = 9.3 Hz). IR (cm^−1^): 3259, 2978, 2934, 2860, 1689, 1621, 1460, 1109, 1032. MS (CV = 20 V): *m*/*z* = 617.3 [M+H]^+^. For C_40_H_57_FN_2_O_2_ (616.89) calcd. C (77.88), H (9.31), F (3.08), N (4.54), found C (77.85), H (9.32), F (3.09), N (4.51). Melting point 147–148 °C.

### 3.5. Cell Cultures and Cytotoxicity Screening Tests

The screening human cancer cell lines used in this investigation were CEM (T-lymphoblastic leukemia), MCF7 (breast carcinoma), HeLa (cervical carcinoma) and G-361 (malignant melanoma) from the European Collection of Authenticated Cell Cultures (Salisbury, UK). Human foreskin fibroblasts (BJ) were used as reference cells, and they were a kind gift from Prof. Bartek from Danish Cancer Society Center (Copenhagen, Denmark). Cells were cultured in DMEM (Dulbecco’s Modified Eagle Medium) or in RPMI 1640 medium (CEM). Media used were supplemented with 10% fetal or 20% (CEM) bovine serum, l-glutamine (2 mM), and 1% penicillin-streptomycin mixture (all from Sigma, MO, USA). The cell lines were maintained under standard cell culture conditions at 37 °C and 5% CO_2_ in a humid environment. Cells were subcultured twice or three times a week using the standard trypsinization procedure. A description of the experimental procedure used in the cytotoxicity screening tests was already published [42,43]. The cell viability was measured after 72 h using resazurin (Sigma, St. Louis, MO, USA). The IC_50_ values (µM) obtained with the compounds 3a–4c are shown in Table 1.

### 3.6. Cell Cycle and Apoptosis

A flow cytometric analysis, SDS–polyacrylamide gel electrophoresis and immunoblotting, and activities of caspase-3/7 were used to detect apoptosis in cervical carcinoma cells (HeLa) and malignant melanoma cells (G-361) treated for 24 h with 3a–3c (*c* = 3, 10 and 30 µM). The DNA content of HeLa or G-361 treated with 3a–3c for 24 h were analyzed using flow cytometry. Cells (HeLa and G-361), treated with the same concentrations of compounds for 24 h, were analyzed using electrophoresis and Western blotting, and the activity of caspase-3/7 by the standard methods. All experimental details of these methods were described in our recent paper [36].

### 3.7. UV Spectrometry as a Tool for Supramolecular Self-Assembly Studies

UV spectra were measured on a Specord 210 spectrometer (Jena Analytical, Jena, Germany) in the wavelength range of 200–400 nm. The stock solutions of the studied compounds were prepared in methanol at a concentration *c* = 1 mg·mL^−1^. A series of methanol/water mixtures were then prepared starting from methanol/water ratio 100/0 up to 0/100 in 20% steps. The stock solutions of the studied compounds (0.15 mL) were added separately to each vial containing methanol/water mixtures (3 mL), and the UV spectra recorded in the wavelength range of 200–400 nm. Spectra were recorded after 1, 4 and 24 h, and then every 24 h for 4 days. The resulting sets of spectra were worked-up and the most pronounced examples of the observed changes are shown in the Supplementary Material.

### 3.8. Statistical Analysis

The data shown are means ± standard deviation (SD) obtained from three independent experiments performed in triplicates. The differences between control and treated cells were analyzed by t-test with a Bonferroni correction using Microsoft Excel 2010 (Microsoft Inc., Redmont, WA, USA). Significance is indicated with an asterisk (*p* ˂ 0.05).

## 4. Conclusions

Oleanolic acid (1), its (3β)-3-acetyloxy derivative 2 and six target compounds 3a–4c were tested for in vitro cytotoxicity in four human cancer cell lines and in normal fibroblasts. The most active compounds 3a–3c identified in this screening were evaluated for induction of apoptosis in cancer cells. They showed a strong induction of apoptosis in cervical carcinoma and malignant melanoma cells after 24 h. The compounds 3a–4c are novel compounds (as far as the literature data were consulted [28,29,30,31,32,33,34,35]) in which structural modifications resulted in modification of the biological effects of their parent components (adaptogens). Amides 3a–4c display cytotoxicity and antiproliferative activity, and cause cell apoptosis in human cervical carcinoma (HeLa) and human malignant melanoma (G-361) cancer cells. The incidence of apoptosis by 3a–3c after 24 h in HeLa cells measured by flow cytometry is in a good correlation with the results from the Western blotting and caspase activity where apoptosis was confirmed. Both cell lines analyzed by Western blotting (HeLa and G-361) showed strong induction of apoptosis after 24 h of treatment with 3a–3c. The most active compounds were 3a and 3b, while 3c had lower activity than 3a and 3b. The compounds 3a–4c show no potential of effect on central nervous system that may indicate potential adaptogen (cf. Table 2). We had already observed modification of the adaptogen effect of parent compounds several times, with diosgenin–betulinic acid conjugates [44] and with oleanolic acid–spermine conjugates [35].

Introductory experiments based on the UV spectra measurements resulted in the finding that several compounds of this series self-assemble into supramolecular systems in methanol/water mixtures. The irregularity in the UV spectra measured within 96 h in the given time intervals was pronounced with the compound 3b in the most remarkable way. Nevertheless, supramolecular self-assembly was proven with the compounds 3a and 3c as well. Because the structures of 3a–3c differ only in the absence or presence and location of the fluorine substituent, we assume that the location of the fluorine atom in 3b might be the main reason for a distinct shape of its UV spectra showing its distinct supramolecular characteristics in comparison with those of 3a and 3c.

## Figures and Tables

**Table 1:** Cytotoxicity screening tests (IC_50_ [µM], 72 h).

Compound	MW ^a^	Cytotoxicity (IC_50_ [µM], 72 h) ^b^					Therapeutic Index (TI)	
		CEM	MCF7	HeLa	G-361	BJ	TI (HeLa)	TI (G-361)
**1**	456.70	>50	>50	>50	>50	>50	–	–
**2**	498.74	>50	>50	>50	>50	>50	–	–
**3a**	640.94	>50	>50	8.7 ± 0.4	9.0 ± 0.4	>50	5	5
**3b**	658.93	>50	>50	6.7 ± 1.4	15.9 ± 6.6	>50	7	3
**3c**	658.93	>50	>50	12.2 ± 4.7	>50	>50	4	–
**4a**	598.90	>50	>50	>50	>50	>50	–	–
**4b**	616.89	>50	>50	27.9 ± 0.8	>50	>50	2	–
**4c**	616.89	>50	13.5 ± 3.3 ^c^	20.3 ± 7.6	>50	>50	2.5	–
CDDP ^d^	300.05	0.8 ± 0.1	7.7 ± 1.7	11.4 ± 3.8	4.5 ± 0.6	6.9 ± 0.9	n.c. ^e^	n.c. ^e^

^a^ MW = molecular weight; ^b^ tryptamine and both fluorotryptamines were inactive in all cancer cell lines and in BJ (IC_50_ > 50 µM); ^c^ TI = 4; ^d^ *cis*-diamminedichloridoplatinum(II) (cisplatin), a pharmacologically used agent for treating cancers, a positive reference compound; ^e^ n.c. = not calculated.

**Table 2:** Physico-chemical and ADME parameters of the target compounds 1, 2 and 3a–4c calculated using the ACD/iLabs software [37].

Compd. or Ref. No.	MW	Physico-Chemical and ADME Parameters ^a^									
		Log *P*	Log *D* (pH 7.4)	Log *S* (pH 7.4)	H_acc_/H_don_/n.m.b.	Bioav. [%]	Log *PS* ∗ *f_u, brain_*	Log *PS*	Log *PB*	Log *BB*	PPB [%]
**1**	456.70	+7.84	+5.35	−3.76	3/2/1	30−70	−5.5	−3.7	−0.41	−0.19	99.39
**2**	498.74	+8.55	+6.05	−4.64	4/1/3	<30	−6.0	−4.2	−0.48	−0.19	99.49
**3a**	640.94	+10.67	+10.67	−9.07	5/2/6	<30	−6.9	−4.6	−0.70	−0.70	99.90
**3b**	658.93	+10.68	+10.68	−9.50	5/2/6	<30	−7.0	−4.6	−0.62	−0.62	99.88
**3c**	658.93	+10.68	+10.68	−9.40	5/2/6	<30	−7.0	−4.6	−0.62	−0.62	99.88
**4a**	598.90	+9.89	+9.89	−8.20	4/3/4	30−70	−6.4	−4.1	−0.58	−0.58	99.87
**4b**	616.89	+10.09	+10.09	−8.70	4/3/4	30−70	−6.6	−4.3	−0.55	−0.55	99.86
**4c**	616.89	+10.09	+10.09	−8.60	4/3/4	30−70	−6.6	−4.3	−0.55	−0.55	99.86
Recom. range	180/500	−0.4/+5.6	-	−6.5/+0.5	max.10/max. 5/−	−	−	−	−1.5/+1.5	−3.0/+1.2	

^a^ log *P*—partition coefficient; log *D*—distribution coefficient; log *S*—predicted aqueous solubility; bioav. = bioavailability—the degree of availability of a chemical by the target tissue; log *PS* ∗ *f_u, brain_*—the brain/plasma equilibration rate, the parameter that is a mathematical modeling parameter based on time required for reaching brain equilibrium; log *PS*—logarithm of the permeability-surface area coefficient; log *PB*—the extent of brain penetration parameter; log *BB*—a hybrid parameter determined by permeability, plasma and brain tissue binding, and active transport mechanism; PPB—plasma protein binding; H_acc_/H_don_/n.m.b. = number of H-bond acceptors/number of H-bond donors/number of movable bonds.

## Supplementary Materials

The following material is available online at https://www.mdpi.com/article/10.3390/plants10102082/s1: NMR spectra of the prepared compounds, pharmacological activity and supramolecular self-assembly relationships, additional data on apoptosis, in silico calculated physico-chemical and ADME parameters, and selected results of investigation of supramolecular self-assembly by UV spectroscopy. Figure S1A: Flow cytometric analysis of cell cycle of G-361 cancer cells. Figure S1B: Apoptosis of G-361 cancer cells. Figure S2: The level of protein markers of apoptosis. Figure S3: Activity of caspase-3/7 after 24 h treatment with 3a–3c (3, 10 and 30 µM) in G-361 cells. Figure S4: Graphs of CVS active/inactive drugs. Figure S5: UV spectra of the selected compounds studied. Graph S1: Blotted membranes with proteins colored by Ponceau-S.
