# Supplementary material for: Novel Oleanolic Acid-Tryptamine and -Fluorotryptamine Amides: From Adaptogens to Agents Targeting In Vitro Cell Apoptosis
Source: Plants (Basel). 2021 Sep 30;10(10):2082. doi: 10.3390/plants10102082 (PMC8540097; doi:10.3390/plants10102082)

SUPPLEMENTARY MATERIAL

Novel Oleanolic Acid–Tryptamine and –Fluorotryptamine Amides: From Adaptogens to Agents Targeting *in vitro* Cell Apoptosis

Uladzimir Bildziukevich,[1,2] Marie Kvasnicová,[3,4] David Šaman,[5] Lucie Rárová,*[,3]
and Zdeněk Wimmer *[,1,2]

[1] Institute of Experimental Botany of the Czech Academy of Sciences, Isotope Laboratory, Vídeňská 1083, CZ-14220 Prague 4; vmagius@gmail.com; wimmer@biomed.cas.cz

[2] University of Chemistry and Technology in Prague, Department of Chemistry of Natural Compounds, Technická 5, CZ-16628 Prague 6; vmagius@gmail.com; wimmerz@vscht.cz

[3] Department of Experimental Biology, Faculty of Science, Palacký University, Šlechtitelů 27, CZ-78371 Olomouc, Czech Republic; kvasnicova@ueb.cas.cz; lucie.rarova@upol.cz;

[4] Laboratory of Growth Regulators, Institute of Experimental Botany of the Czech Academy of Sciences, and Faculty of Science, Palacký University, Šlechtitelů 27, CZ-78371 Olomouc, Czech Republic; kvasnicova@ueb.cas.cz;

[5] Institute of Organic Chemistry and Biochemistry of the Czech Academy of Sciences, Flemingovo náměstí 2, CZ-16610 Prague 6; nmrsaman@gmail.com

Table of Content

1. NMR spectra of the prepared compounds S3

2. Pharmacological activity and supramolecular self-assembly S16

3. Apoptosis in human cancer cells S18

4. *In silico* calculated physico-chemical and ADME parameters S20

5. Investigation of supramolecular self-assembly by UV spectroscopy S23

6. Blotted membranes with proteins colored by Ponceau-S S31

1. NMR spectra of the prepared compounds.

1.1. (3β)-3-(Acetyloxy)olean-12-en-28-oic acid (**2**)

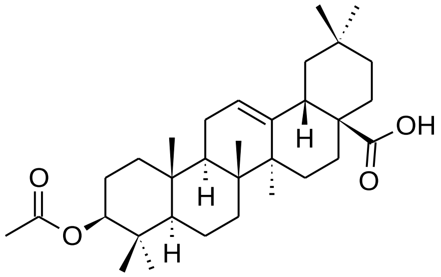

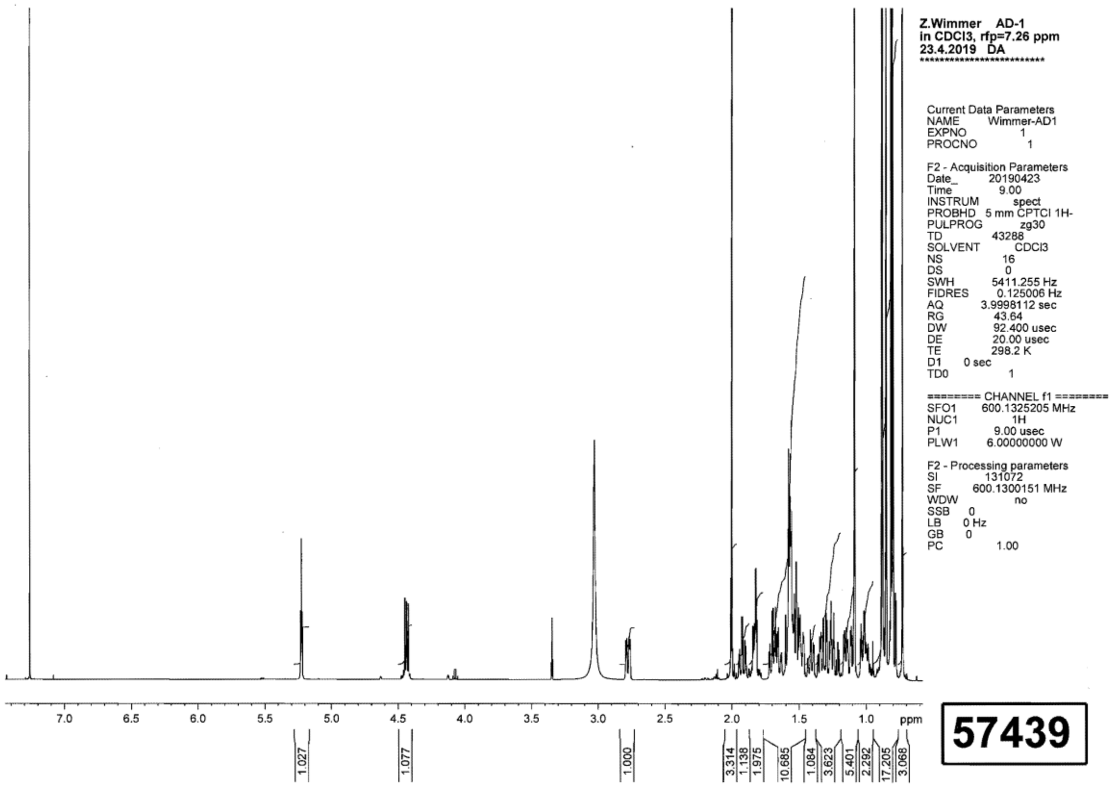

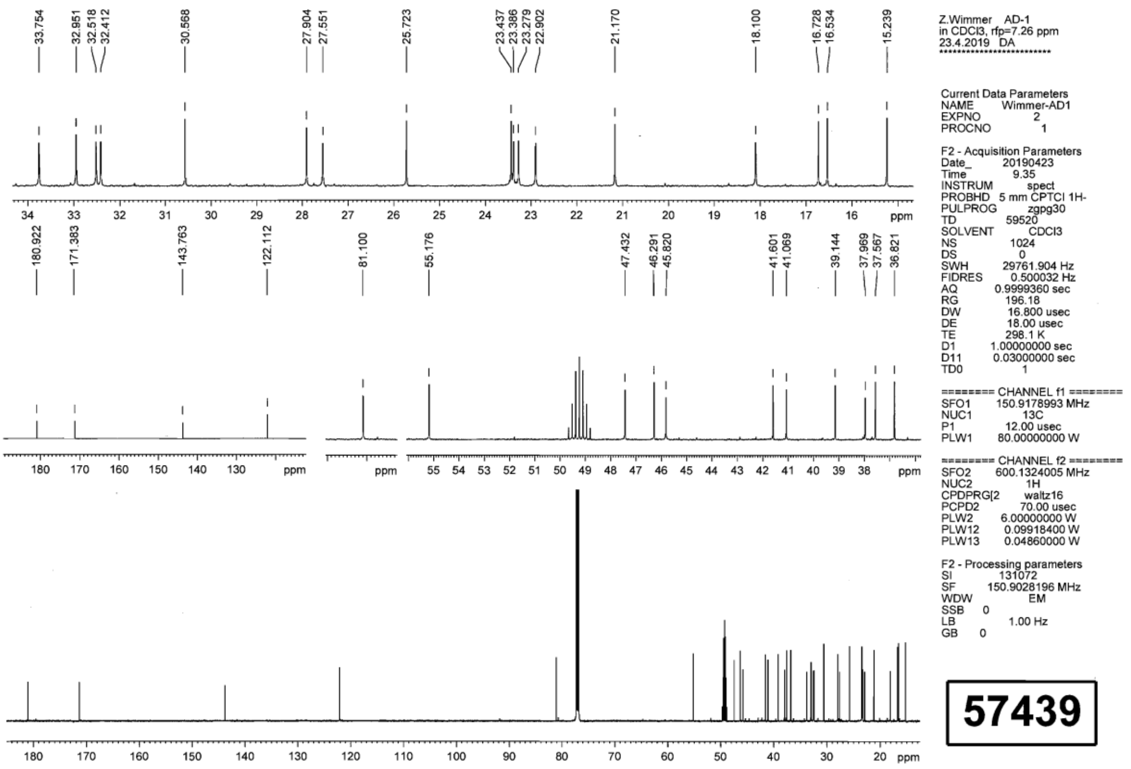

1.2. (3β)-28-{[2-(1*H*-indol-3-yl)ethyl]amino-28-oxoolean-12-en-3-yl-acetate (**3a**)

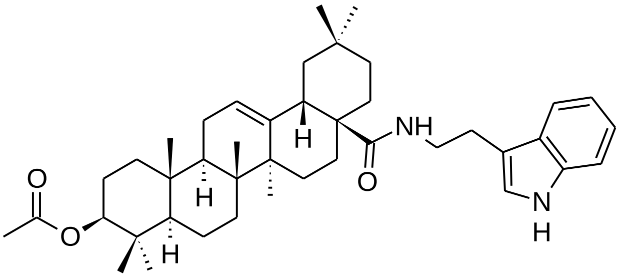

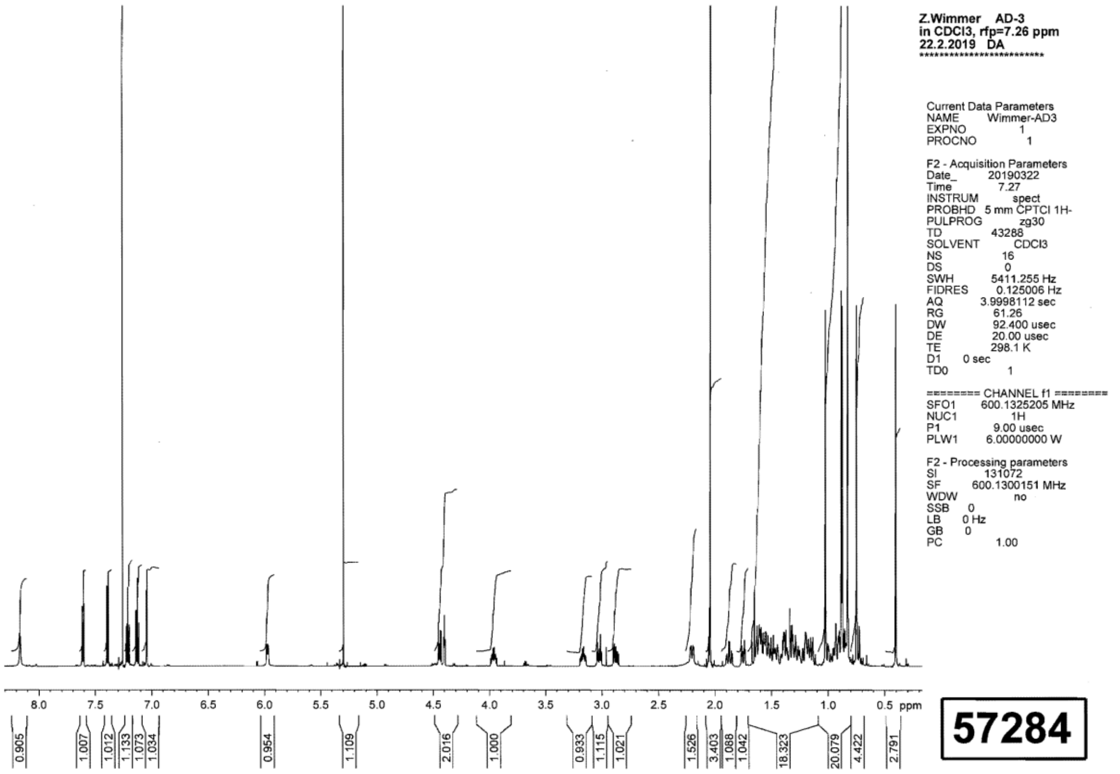
Z.Wimmer AD-3
in CDCl3, rfp=7.26 ppm
22.2.2019 DA
Current Data Parameters
NAME Wimmer-AD3
EXPNO 1
PROCNO 1
F2 - Acquisition Parameters
Date_ 20190322
Time 7.27
INSTRUM spect
PROBHD 5 mm CPTCI 1H-
PULPROG zg30
TD 43288
SOLVENT CDCl3
NS 16
DS 0
SWH 5411.255 Hz
FIDRES 0.125006 Hz
AQ 3.9998112 sec
RG 61.26
DW 92.400 usec
DE 20.00 usec
TE 298.1 K
D1 0 sec
TD0 1
======== CHANNEL f1 ========
SFO1 600.1325205 MHz
NUC1 1H
P1 9.00 usec
PLW1 6.00000000 W
F2 - Processing parameters
SI 131072
SF 600.1300151 MHz
WDW no
SSB 0
LB 0 Hz
GB 0
PC 1.00
57284

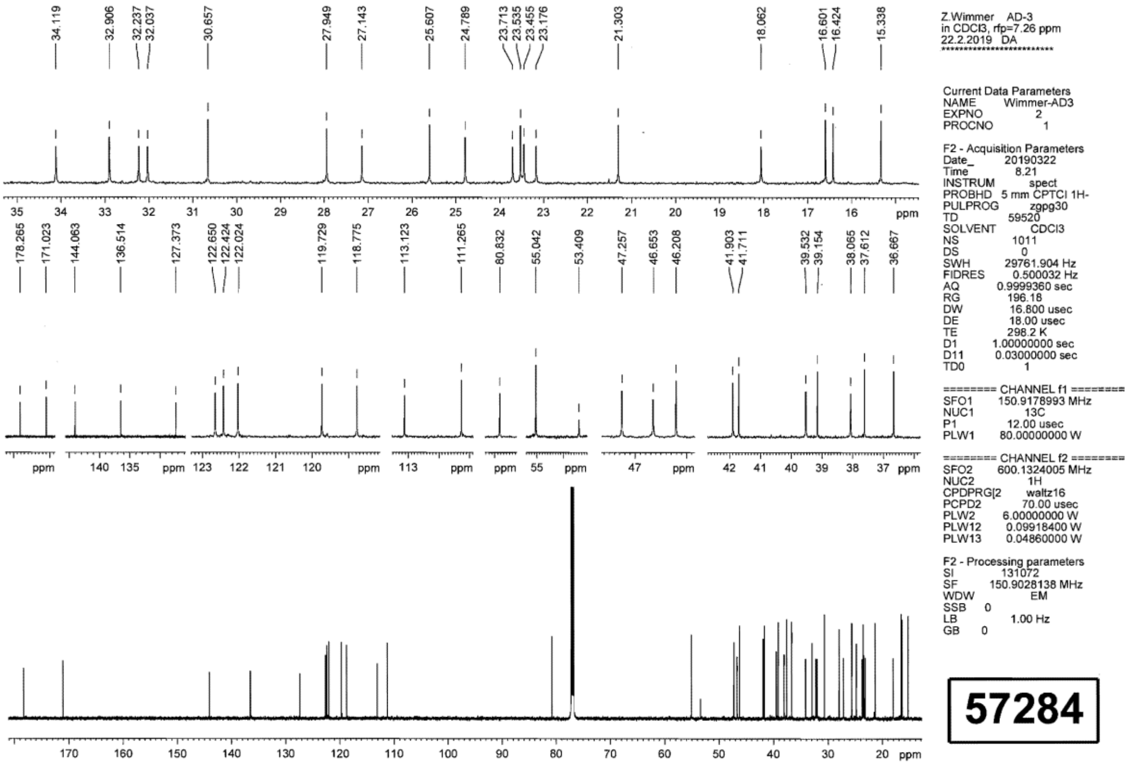
Z.Wimmer AD-3
in CDCl3, rfp=7.26 ppm
22.2.2019 DA
Current Data Parameters
NAME Wimmer-AD3
EXPNO 2
PROCNO 1
F2 - Acquisition Parameters
Date_ 20190322
Time 8.21
INSTRUM spect
PROBHD 5 mm CPTCI 1H-
PULPROG zgpg30
TD 59520
SOLVENT CDCl3
NS 1011
DS 0
SWH 29761.904 Hz
FIDRES 0.500032 Hz
AQ 0.9999360 sec
RG 196.18
DW 16.800 usec
DE 18.00 usec
TE 298.2 K
D1 1.00000000 sec
D11 0.03000000 sec
TD0 1
======== CHANNEL f1 ========
SFO1 150.9178993 MHz
NUC1 13C
P1 12.00 usec
PLW1 80.00000000 W
======== CHANNEL f2 ========
SFO2 600.1324005 MHz
NUC2 1H
CPDPRG[2 waltz16
PCPD2 70.00 usec
PLW2 6.00000000 W
PLW12 0.09918400 W
PLW13 0.04860000 W
F2 - Processing parameters
SI 131072
SF 150.9028138 MHz
WDW EM
SSB 0
LB 1.00 Hz
GB 0
57284

1.3. (3β)-28-{[2-(6-fluoro-1*H*-indol-3-yl)ethyl]amino}-28-oxoolean-12-en-3-yl-acetate (**3b**)

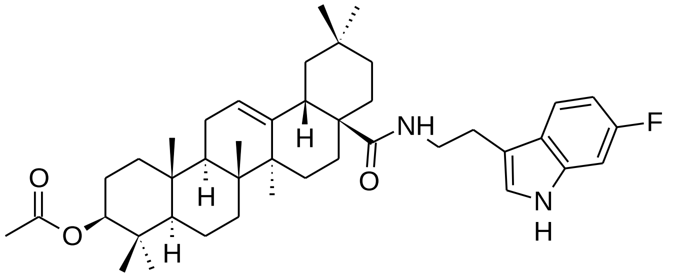

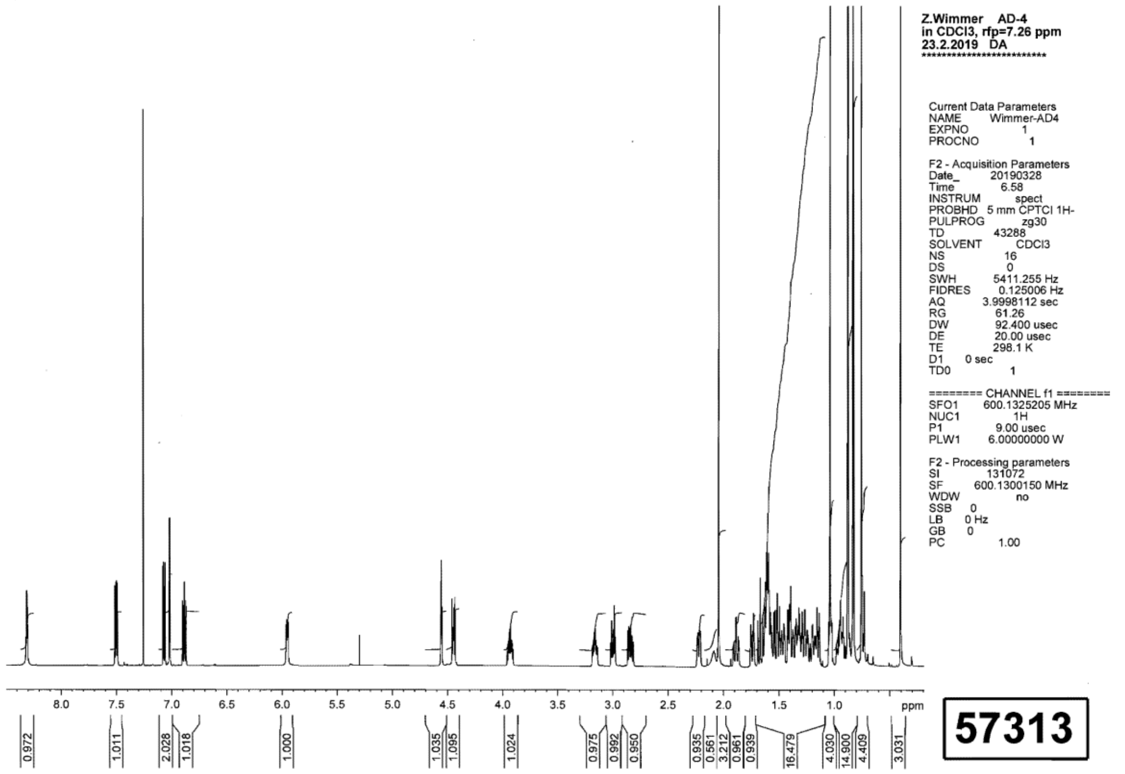

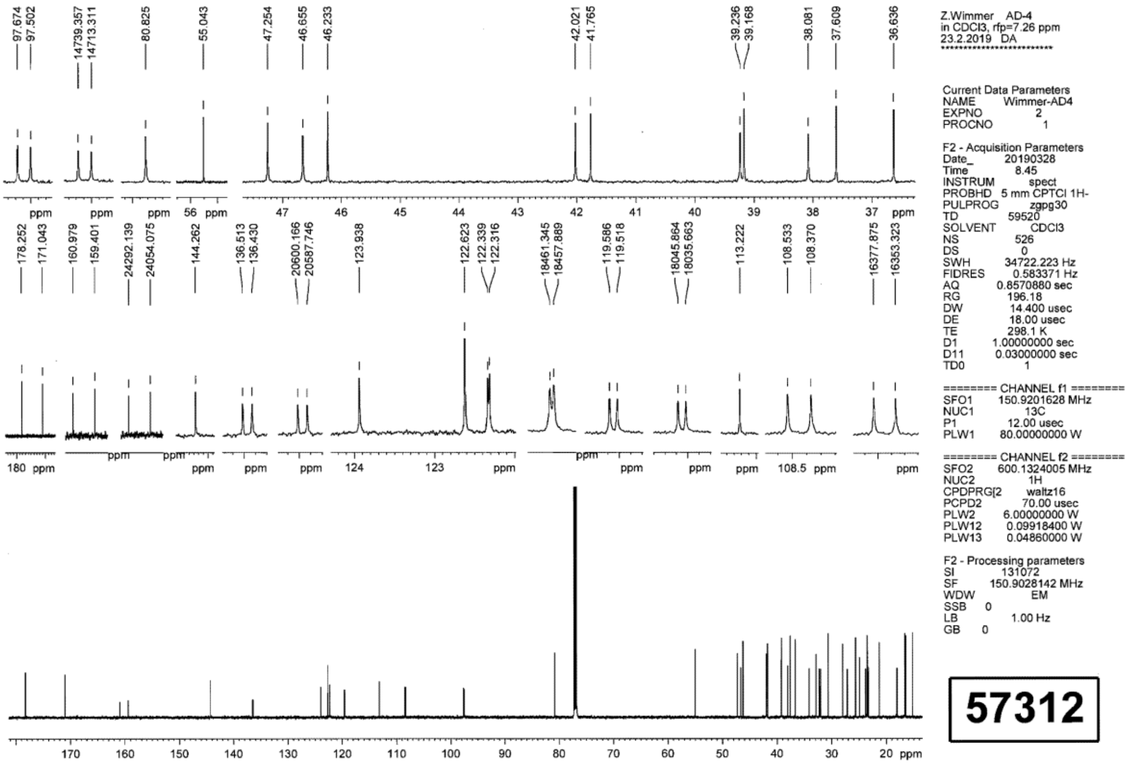
Z.Wimmer AD-4
in CDCl3, rfp=7.26 ppm
23.2.2019 DA
Current Data Parameters
NAME Wimmer-AD4
EXPNO 2
PROCNO 1
F2 - Acquisition Parameters
Date_ 20190328
Time 8.45
INSTRUM spect
PROBHD 5 mm CPTCI 1H-
PULPROG zgpg30
TD 59520
SOLVENT CDCl3
NS 526
DS 0
SWH 34722.223 Hz
FIDRES 0.583371 Hz
AQ 0.8570880 sec
RG 196.18
DW 14.400 usec
DE 18.00 usec
TE 298.1 K
D1 1.00000000 sec
D11 0.03000000 sec
TD0 1
======== CHANNEL f1 ========
SFO1 150.9201628 MHz
NUC1 13C
P1 12.00 usec
PLW1 80.00000000 W
======== CHANNEL f2 ========
SFO2 600.1324005 MHz
NUC2 1H
CPDPRG[2 waltz16
PCPD2 70.00 usec
PLW2 6.00000000 W
PLW12 0.09918400 W
PLW13 0.04860000 W
F2 - Processing parameters
SI 131072
SF 150.9028142 MHz
WDW EM
SSB 0
LB 1.00 Hz
GB 0
57312
ppm

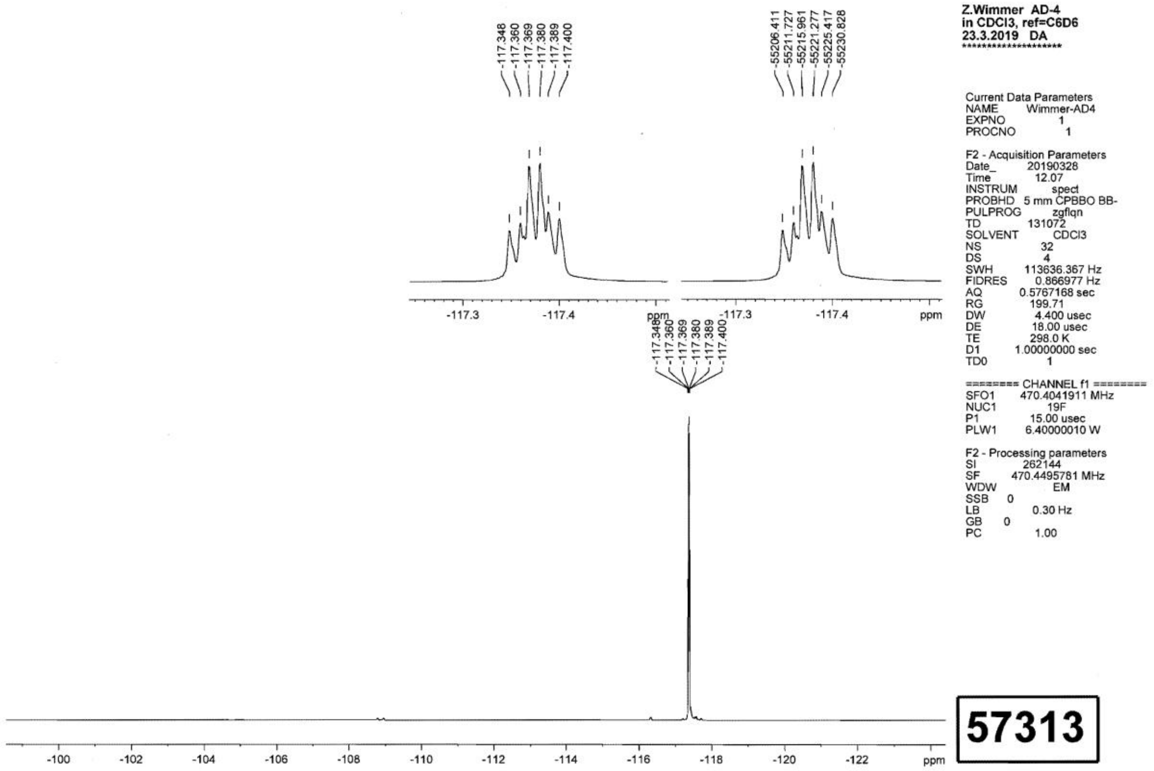
Z.Wimmer AD-4
in CDCl3, ref=C6D6
23.3.2019 DA
Current Data Parameters
NAME Wimmer-AD4
EXPNO 1
PROCNO 1
F2 - Acquisition Parameters
Date_ 20190328
Time 12.07
INSTRUM spect
PROBHD 5 mm CPBBO BB-
PULPROG zgflqn
TD 131072
SOLVENT CDCl3
NS 32
DS 4
SWH 113636.367 Hz
FIDRES 0.866977 Hz
AQ 0.5767168 sec
RG 199.71
DW 4.400 usec
DE 18.00 usec
TE 298.0 K
D1 1.00000000 sec
TD0 1
======== CHANNEL f1 ========
SFO1 470.4041911 MHz
NUC1 19F
P1 15.00 usec
PLW1 6.40000010 W
F2 - Processing parameters
SI 262144
SF 470.4495781 MHz
WDW EM
SSB 0
LB 0.30 Hz
GB 0
PC 1.00
57313
ppm

1.4. (3β)-28-{[2-(5-fluoro-1*H*-indol-3-yl)ethyl]amino}-28-oxoolean-12-en-3-yl-acetate (**3c**)

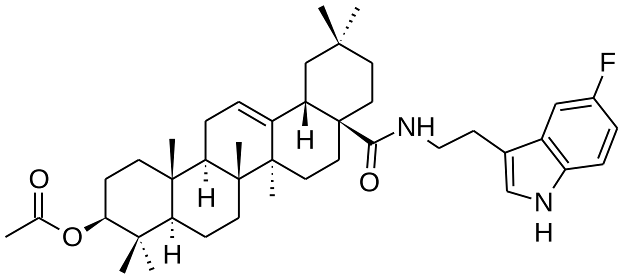

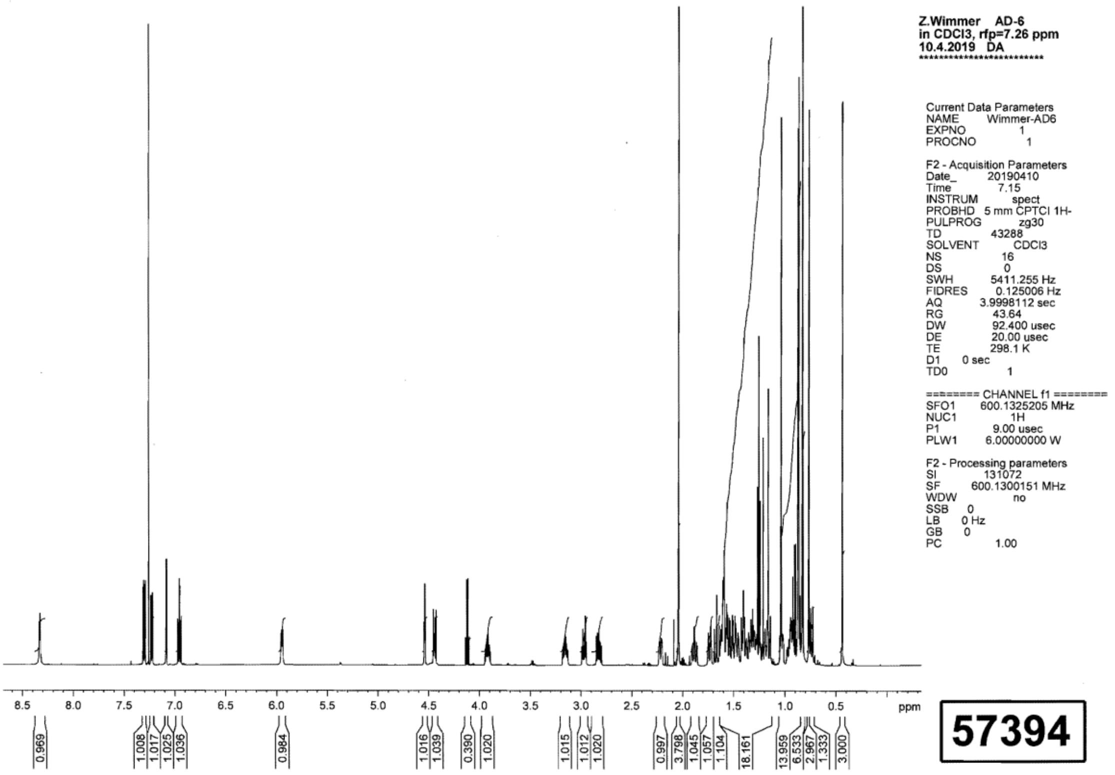

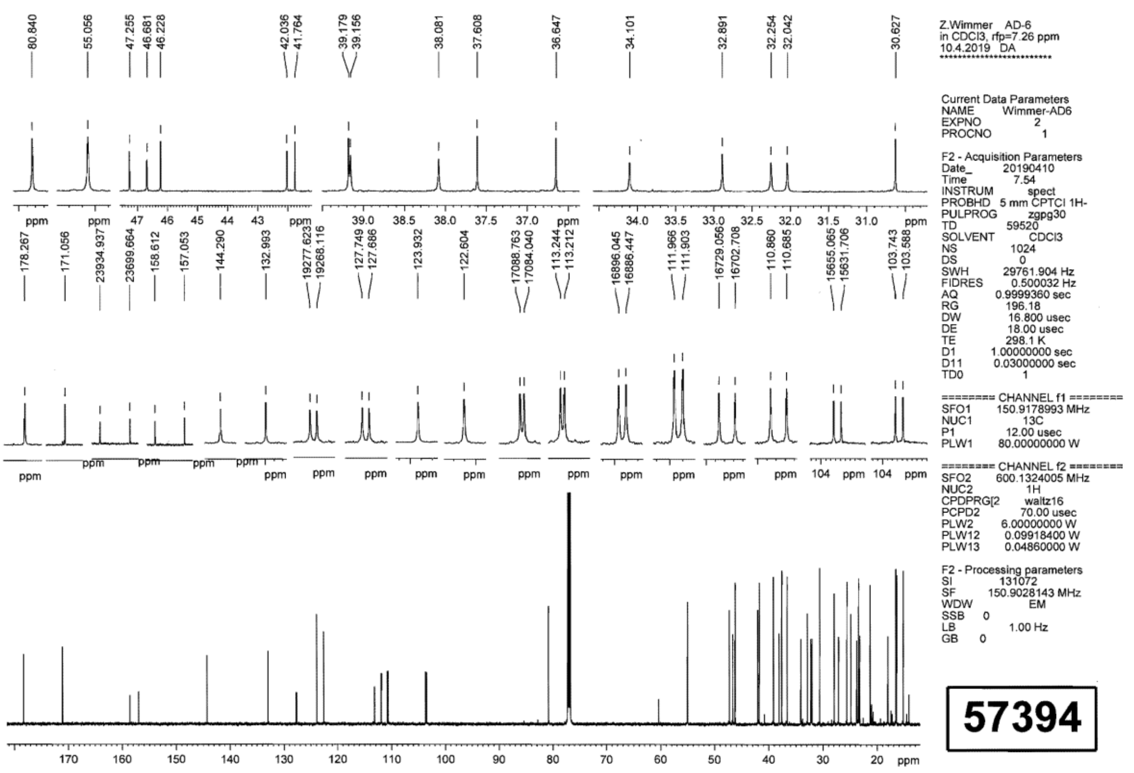
Z.Wimmer AD-6
in CDCl3, rfp=7.26 ppm
10.4.2019 DA
Current Data Parameters
NAME Wimmer-AD6
EXPNO 2
PROCNO 1
F2 - Acquisition Parameters
Date_ 20190410
Time 7.54
INSTRUM spect
PROBHD 5 mm CPTCI 1H-
PULPROG zgpg30
TD 59520
SOLVENT CDCl3
NS 1024
DS 0
SWH 29761.904 Hz
FIDRES 0.500032 Hz
AQ 0.9999360 sec
RG 196.18
DW 16.800 usec
DE 18.00 usec
TE 298.1 K
D1 1.00000000 sec
D11 0.03000000 sec
TD0 1
======== CHANNEL f1 ========
SFO1 150.9178993 MHz
NUC1 13C
P1 12.00 usec
PLW1 80.00000000 W
======== CHANNEL f2 ========
SFO2 600.1324005 MHz
NUC2 1H
CPDPRG[2 waltz16
PCPD2 70.00 usec
PLW2 6.00000000 W
PLW12 0.09918400 W
PLW13 0.04860000 W
F2 - Processing parameters
SI 131072
SF 150.9028143 MHz
WDW EM
SSB 0
LB 1.00 Hz
GB 0
57394

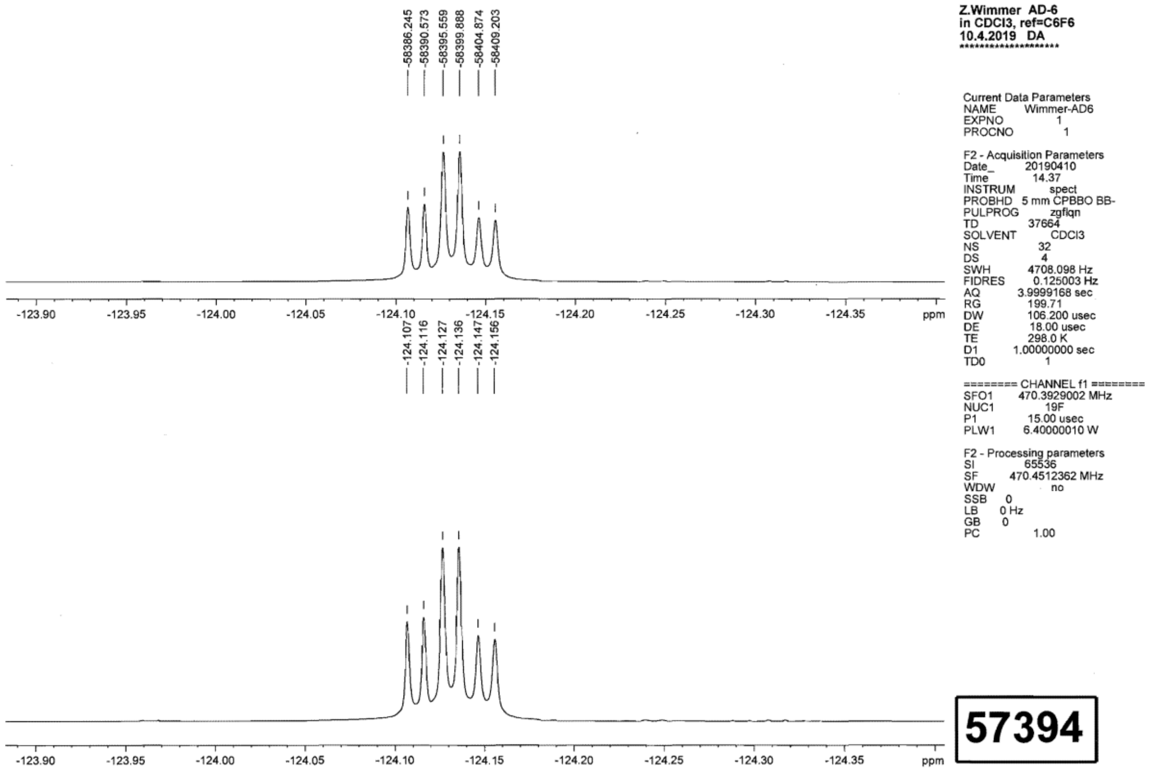
Z.Wimmer AD-6
in CDCl3, ref=C6F6
10.4.2019 DA
-58386.245
-58390.573
-58395.559
-58399.888
-58404.874
-58409.203
-124.107
-124.116
-124.127
-124.136
-124.147
-124.156
Current Data Parameters
NAME Wimmer-AD6
EXPNO 1
PROCNO 1
F2 - Acquisition Parameters
Date_ 20190410
Time 14.37
INSTRUM spect
PROBHD 5 mm CPBBO BB-
PULPROG zgflqn
TD 37664
SOLVENT CDCl3
NS 32
DS 4
SWH 4708.098 Hz
FIDRES 0.125003 Hz
AQ 3.9999168 sec
RG 199.71
DW 106.200 usec
DE 18.00 usec
TE 298.0 K
D1 1.00000000 sec
TD0 1
======== CHANNEL f1 ========
SFO1 470.3929002 MHz
NUC1 19F
P1 15.00 usec
PLW1 6.40000010 W
F2 - Processing parameters
SI 65536
SF 470.4512362 MHz
WDW no
SSB 0
LB 0 Hz
GB 0
PC 1.00
57394

1.5. (3β)-3-hydroxy-*N*-[2-(1*H*-indol-3-yl)ethyl]olean-12-en-28-amide (**4a**)

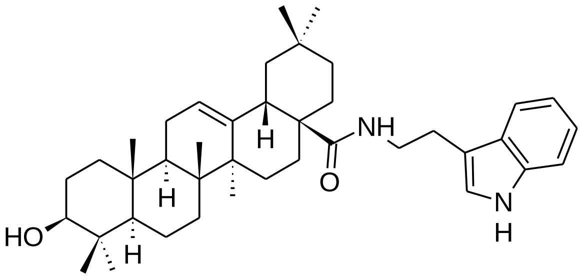

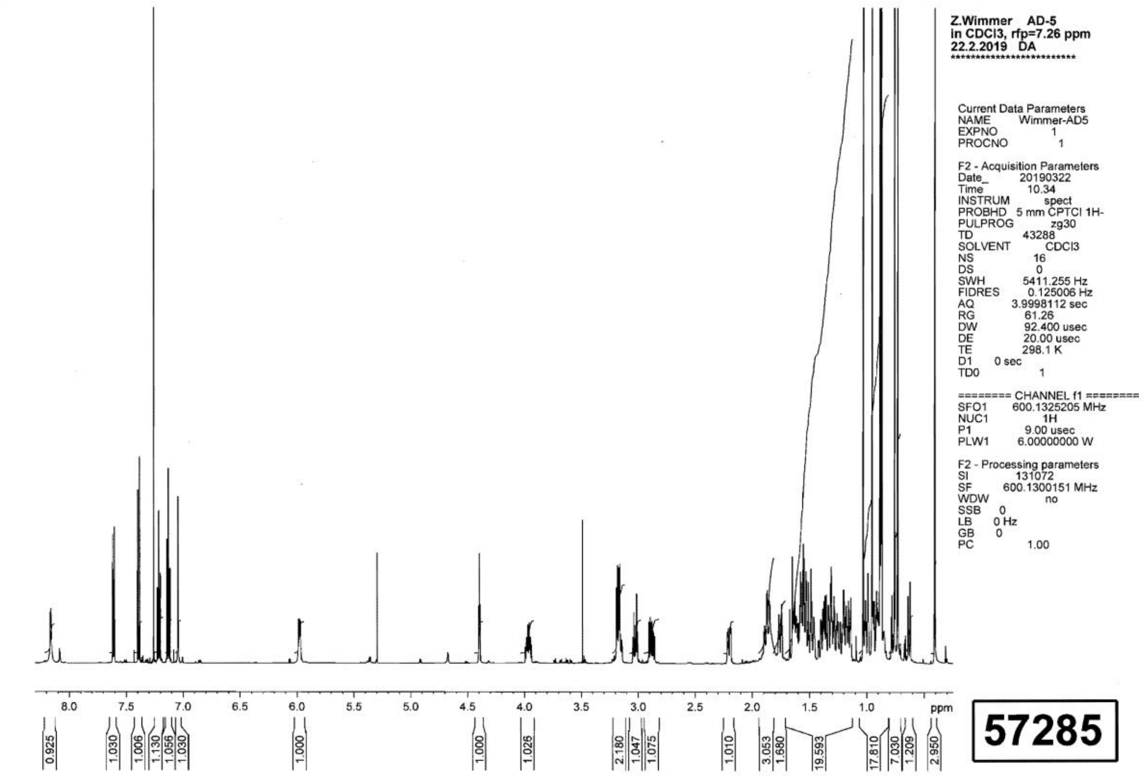

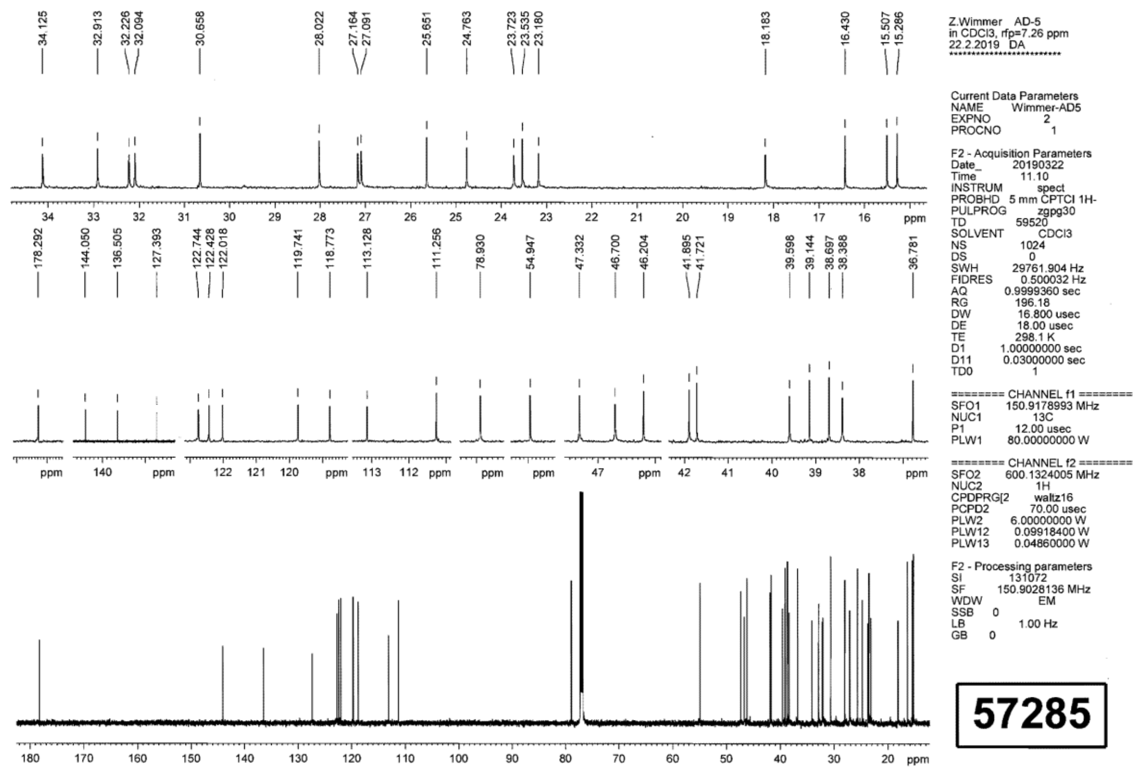
34.125 32.913 32.226 32.094 30.658 28.022 27.164 27.091 25.651 24.763 23.723 23.535 23.180 18.183 16.430 15.507 15.286
178.292 144.050 136.505 127.393 122.744 122.428 122.018 119.741 118.773 113.128 111.256 78.930 54.947 47.332 46.700 46.204 41.895 41.721 39.598 39.144 38.697 38.388 36.781
Z.Wimmer AD-5
in CDCl3, rfp=7.26 ppm
22.2.2019 DA
Current Data Parameters
NAME Wimmer-AD5
EXPNO 2
PROCNO 1
F2 - Acquisition Parameters
Date_ 20190322
Time 11.10
INSTRUM spect
PROBHD 5 mm CPTCI 1H-
PULPROG zgpg30
TD 59520
SOLVENT CDCl3
NS 1024
DS 0
SWH 29761.904 Hz
FIDRES 0.500032 Hz
AQ 0.9999360 sec
RG 196.18
DW 16.800 usec
DE 18.00 usec
TE 298.1 K
D1 1.00000000 sec
D11 0.03000000 sec
TD0 1
======== CHANNEL f1 ========
SFO1 150.9178993 MHz
NUC1 13C
P1 12.00 usec
PLW1 80.00000000 W
======== CHANNEL f2 ========
SFO2 600.1324005 MHz
NUC2 1H
CPDPRG[2 waltz16
PCPD2 70.00 usec
PLW2 6.00000000 W
PLW12 0.09918400 W
PLW13 0.04860000 W
F2 - Processing parameters
SI 131072
SF 150.9028136 MHz
WDW EM
SSB 0
LB 1.00 Hz
GB 0
57285
ppm

1.6. (3β)-*N*-[2-(6-fluoro-1*H*-indol-3-yl)ethyl]-3-hydroxyolean-12-en-28-amide (**4b**)

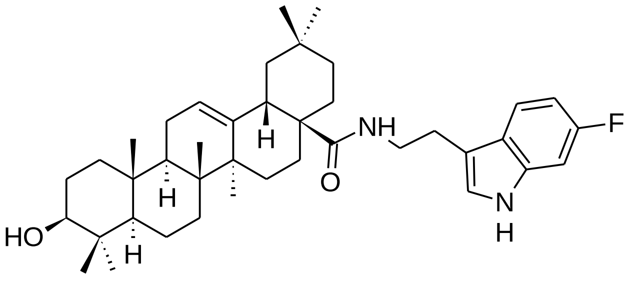

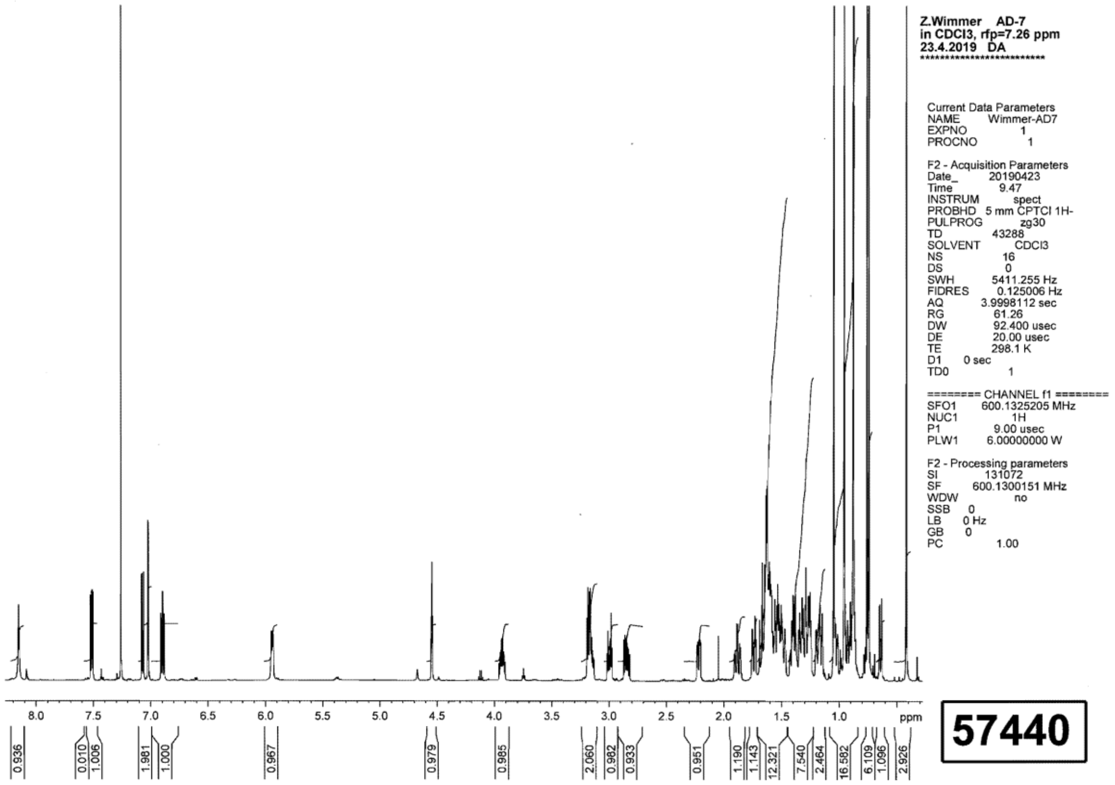

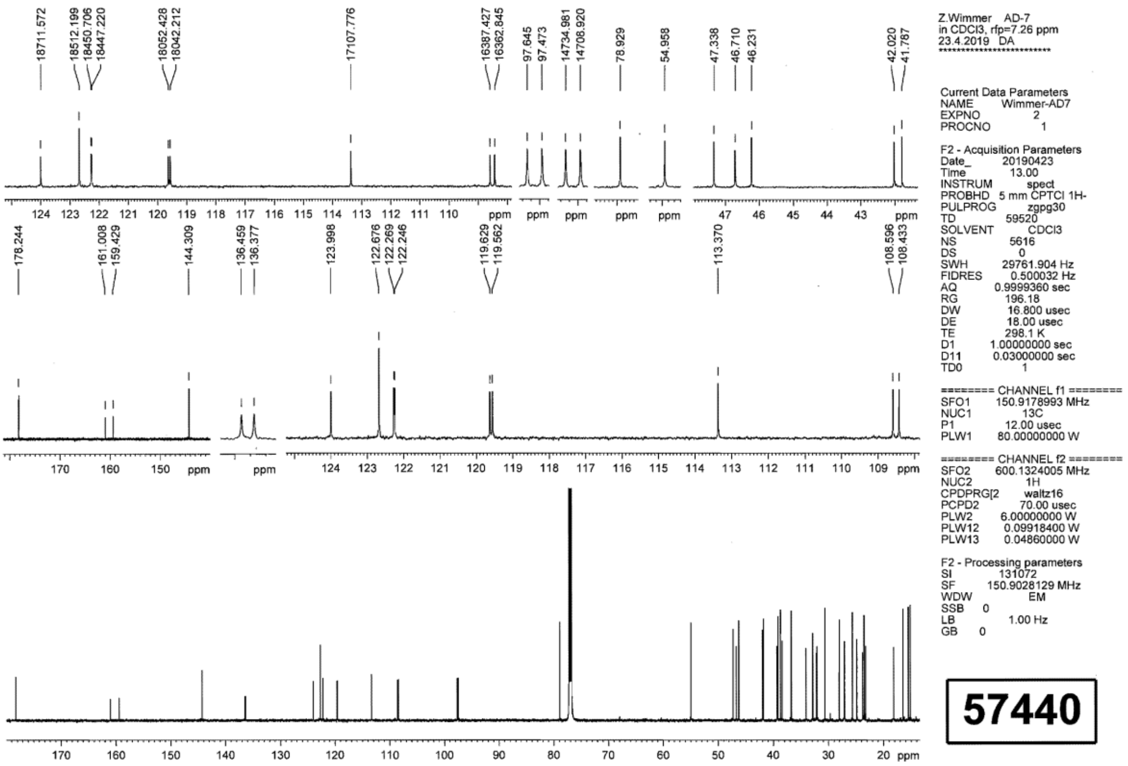
Z.Wimmer AD-7
in CDCl3, rfp=7.26 ppm
23.4.2019 DA
Current Data Parameters
NAME Wimmer-AD7
EXPNO 2
PROCNO 1
F2 - Acquisition Parameters
Date_ 20190423
Time 13.00
INSTRUM spect
PROBHD 5 mm CPTCI 1H-
PULPROG zgpg30
TD 59520
SOLVENT CDCl3
NS 5616
DS 0
SWH 29761.904 Hz
FIDRES 0.500032 Hz
AQ 0.9999360 sec
RG 196.18
DW 16.800 usec
DE 18.00 usec
TE 298.1 K
D1 1.00000000 sec
D11 0.03000000 sec
TD0 1
======== CHANNEL f1 ========
SFO1 150.9178993 MHz
NUC1 13C
P1 12.00 usec
PLW1 80.00000000 W
======== CHANNEL f2 ========
SFO2 600.1324005 MHz
NUC2 1H
CPDPRG[2 waltz16
PCPD2 70.00 usec
PLW2 6.00000000 W
PLW12 0.09918400 W
PLW13 0.04860000 W
F2 - Processing parameters
SI 131072
SF 150.9028129 MHz
WDW EM
SSB 0
LB 1.00 Hz
GB 0
57440

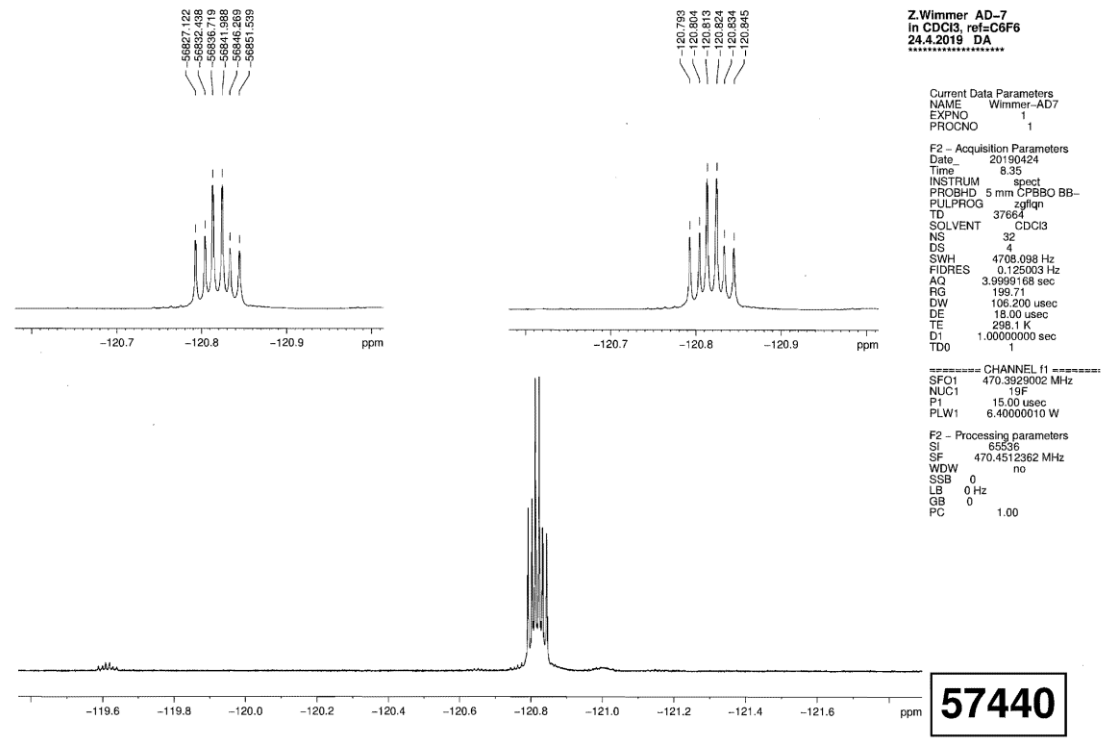
Z.Wimmer AD–7
in CDCl3, ref=C6F6
24.4.2019 DA
Current Data Parameters
NAME Wimmer–AD7
EXPNO 1
PROCNO 1
F2 – Acquisition Parameters
Date_ 20190424
Time 8.35
INSTRUM spect
PROBHD 5 mm CPBBO BB–
PULPROG zgflqn
TD 37664
SOLVENT CDCl3
NS 32
DS 4
SWH 4708.098 Hz
FIDRES 0.125003 Hz
AQ 3.9999168 sec
RG 199.71
DW 106.200 usec
DE 18.00 usec
TE 298.1 K
D1 1.00000000 sec
TD0 1
======== CHANNEL f1 ========
SFO1 470.3929002 MHz
NUC1 19F
P1 15.00 usec
PLW1 6.40000010 W
F2 – Processing parameters
SI 65536
SF 470.4512362 MHz
WDW no
SSB 0
LB 0 Hz
GB 0
PC 1.00
57440

1.7. (3β)-*N*-[2-(5-fluoro-1*H*-indol-3-yl)ethyl]-3-hydroxyolean-12-en-28-amide (**4c**)

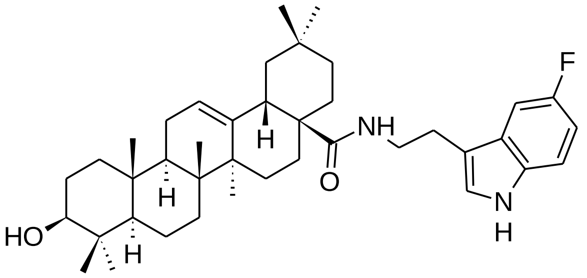

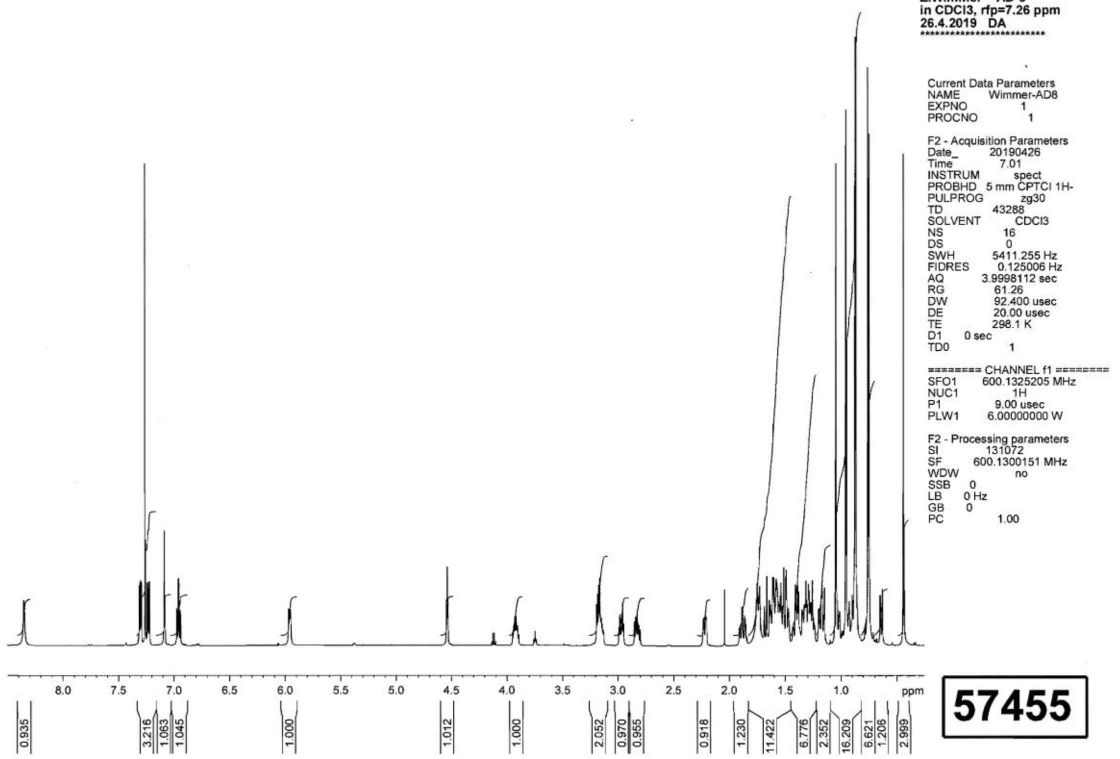

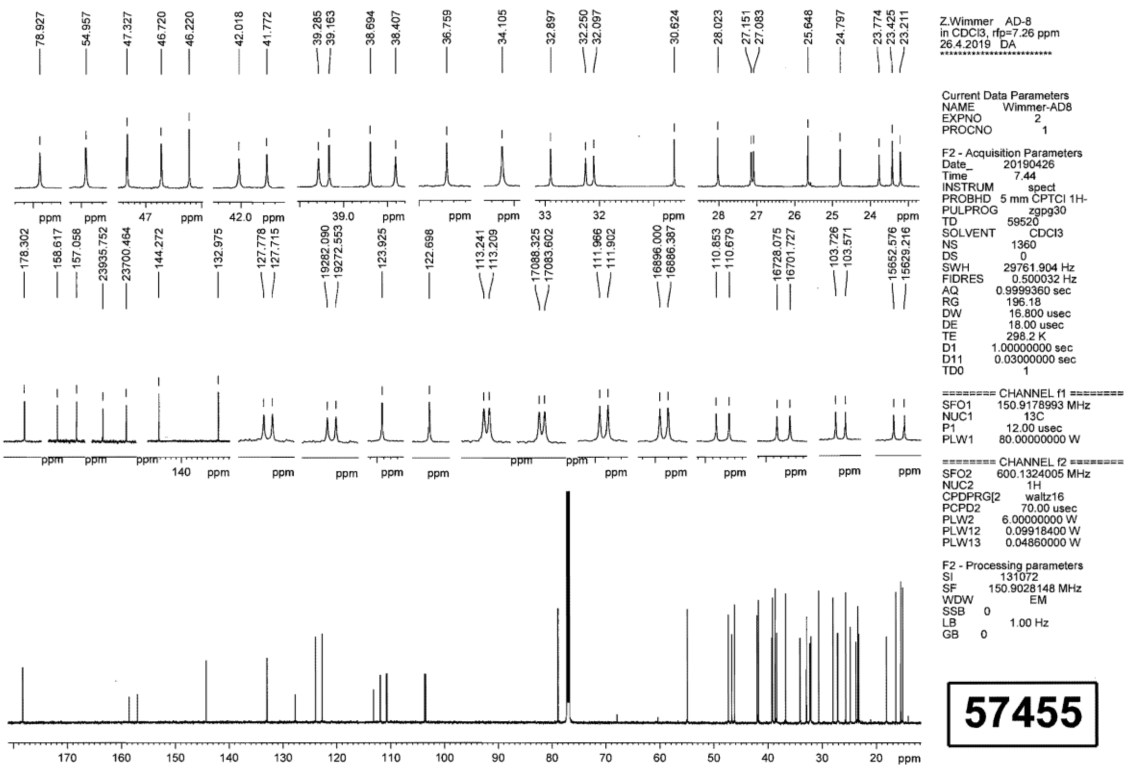
Z.Wimmer AD-8
in CDCl3, rfp=7.26 ppm
26.4.2019 DA
78.927 54.957 47.327 46.720 46.220 42.018 41.772 39.285 39.163 38.694 38.407 36.759 34.105 32.897 32.250 32.097 30.624 28.023 27.151 27.083 25.648 24.797 23.774 23.425 23.211
178.302 158.617 157.058 23935.752 23700.464 144.272 132.975 127.778 127.715 19282.090 19272.553 123.925 122.698 113.241 113.209 17088.325 17083.602 111.966 111.902 16896.000 16886.387 110.853 110.679 16728.075 16701.727 103.726 103.571 15652.576 15629.216
Current Data Parameters
NAME Wimmer-AD8
EXPNO 2
PROCNO 1
F2 - Acquisition Parameters
Date_ 20190426
Time 7.44
INSTRUM spect
PROBHD 5 mm CPTCI 1H-
PULPROG zgpg30
TD 59520
SOLVENT CDCl3
NS 1360
DS 0
SWH 29761.904 Hz
FIDRES 0.500032 Hz
AQ 0.9999360 sec
RG 196.18
DW 16.800 usec
DE 18.00 usec
TE 298.2 K
D1 1.00000000 sec
D11 0.03000000 sec
TD0 1
======== CHANNEL f1 ========
SFO1 150.9178993 MHz
NUC1 13C
P1 12.00 usec
PLW1 80.00000000 W
======== CHANNEL f2 ========
SFO2 600.1324005 MHz
NUC2 1H
CPDPRG[2 waltz16
PCPD2 70.00 usec
PLW2 6.00000000 W
PLW12 0.09918400 W
PLW13 0.04860000 W
F2 - Processing parameters
SI 131072
SF 150.9028148 MHz
WDW EM
SSB 0
LB 1.00 Hz
GB 0
57455
170 160 150 140 130 120 110 100 90 80 70 60 50 40 30 20 ppm

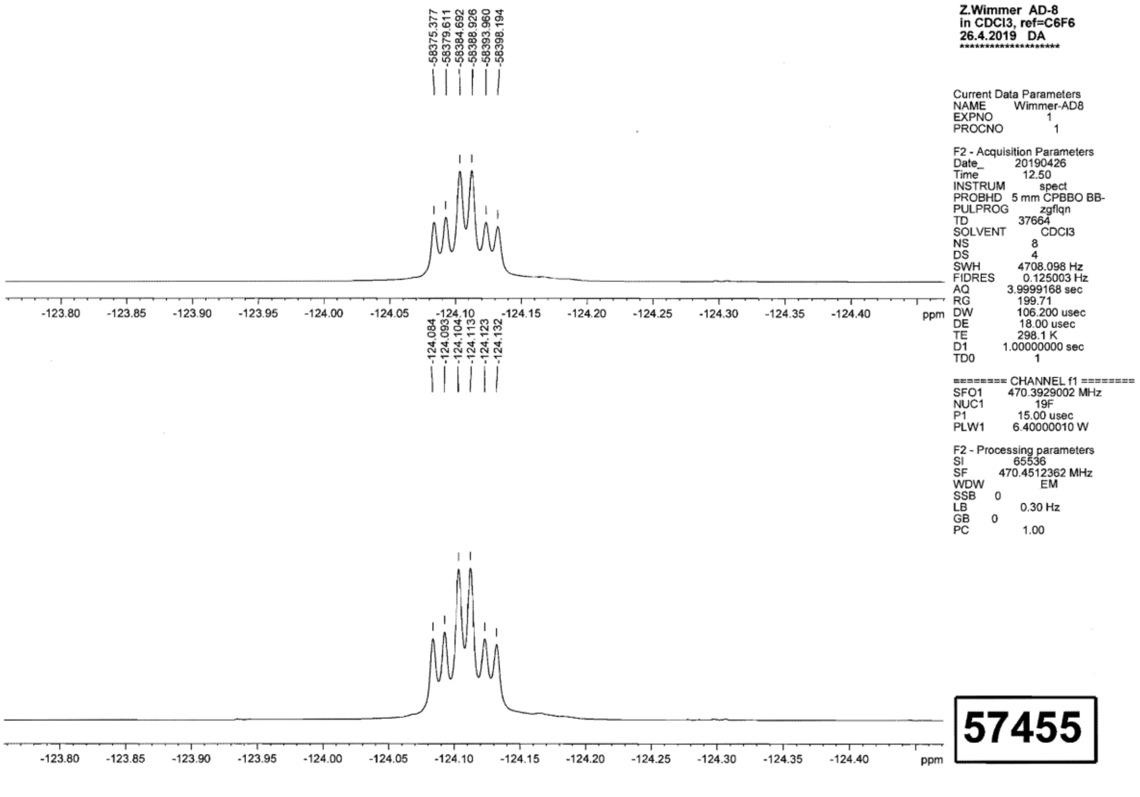
Z.Wimmer AD-8
in CDCl3, ref=C6F6
26.4.2019 DA
-58375.377 -58379.611 -58384.692 -58388.926 -58393.960 -58398.194
-124.084 -124.093 -124.104 -124.113 -124.123 -124.132
Current Data Parameters
NAME Wimmer-AD8
EXPNO 1
PROCNO 1
F2 - Acquisition Parameters
Date_ 20190426
Time 12.50
INSTRUM spect
PROBHD 5 mm CPBBO BB-
PULPROG zgflqn
TD 37664
SOLVENT CDCl3
NS 8
DS 4
SWH 4708.098 Hz
FIDRES 0.125003 Hz
AQ 3.9999168 sec
RG 199.71
DW 106.200 usec
DE 18.00 usec
TE 298.1 K
D1 1.00000000 sec
TD0 1
======== CHANNEL f1 ========
SFO1 470.3929002 MHz
NUC1 19F
P1 15.00 usec
PLW1 6.40000010 W
F2 - Processing parameters
SI 65536
SF 470.4512362 MHz
WDW EM
SSB 0
LB 0.30 Hz
GB 0
PC 1.00
57455
-123.80 -123.85 -123.90 -123.95 -124.00 -124.05 -124.10 -124.15 -124.20 -124.25 -124.30 -124.35 -124.40 ppm

2. Pharmacological activity and supramolecular self-assembly.

The results of cytotoxicity screening tests could be affected by supramolecular self-assembly of several tested compounds (cf. values in italics in **Table S1**). We have already observed such effects recently [4]. Irregularity shown in italics (**Table S1**) indicated that the tested compounds self-assemble during the screening test. Our hypothesis is that self-assembly appears either in the cells or on the cell wall, and spontaneous self-assembly may influence cytotoxicity either in a positive way or in a negative way (see the numbers in italics in **Table S1**). Self-assembly is also indicated by higher standard deviation (SD) values (numbers in bold in **Table S1**). Recently, we have proven that self-assembly is a dynamic process [4]. Supramolecular systems transform into other supramolecular forms depending on the environment. The explanation for the irregularity of cytotoxicity values given in italics (**Table S1**) is analogous as already published [4], however, with these compounds (**3a**–**4c**) is much less pronounced than with the series of compounds published recently [4]. The cancer cells of different types are either more or less sensitive to the possible supramolecular structures that appear in the system. HeLa cells seem to respond more often to the appearance of self-aggregated system than other tested human cancer cells.

Table S1. Cytotoxicity screening tests (IC_{50} [µM], 72 h) with the details from repeated screening runs (numbers in italics).

Compound	MW	Cytotoxicity (IC_{50} [µM], 72 h)				
		CEM	MCF7	HeLa	G361	BJ
**1**	456.70	> 50	> 50	> 50	> 50	> 50
**2**	498.74	> 50	> 50	> 50	> 50	> 50
**3a**	640.94	> 50	> 50	8.7 ± 0.4	9.0 ± 0.4	> 50
				8.4/9.0	*8.7/9.2*	
**3b**	658.93	> 50	> 50	6.7 ± 1.4	**15.9 ± 6.6**	> 50
				7.7/13.1/5.7	*14.9/>50/23.0/9.9*	
**3c**	658.93	> 50	> 50	**12.2 ± 4.7**	> 50	> 50
				10.4/17.6/8.7		
**4a**	598.90	> 50	> 50	> 50	> 50	> 50
				20.4/>50/>50	*19.7/>50/>50*	
**4b**	616.89	> 50	> 50	27.9 ± 0.8	> 50	> 50
			44.5/>50	*28.5/>50/27.3*		
**4c**	616.89	> 50	**13.5 ± 3.3**	**20.3 ± 7.6**	> 50	> 50
			31.8/>50/11.2/15.8	*14.9/29/17.1*		

3. Apoptosis in human cancer cells.

A detailed description of investigation of apoptosis in the HeLa and G-361 cancer cells is given in the main text of this paper. Because the results achieved with the G-361 cancer cells appeared to be less pronounced than those achieved with the HeLa cells, **Figures S1A**, **S1B**, **S2** and **S3** showing the results with G-361 cells are presented in the Supplementary material.

Figure S1A and S1B. Flow cytometric analysis of cell cycle (A) and apoptosis (B) of human malignant melanoma cells (G-361) after 24 h with **3a**–**3c** (3, 10 and 30 μM). The bars 0 means control untreated cells, the bars 0+ means control with DMSO. Error bars are omitted for graph clarity. Experiment was repeated at least twice in duplicates.

Figure S1A. Flow cytometric analysis of cell cycle of G-361 cancer cells.

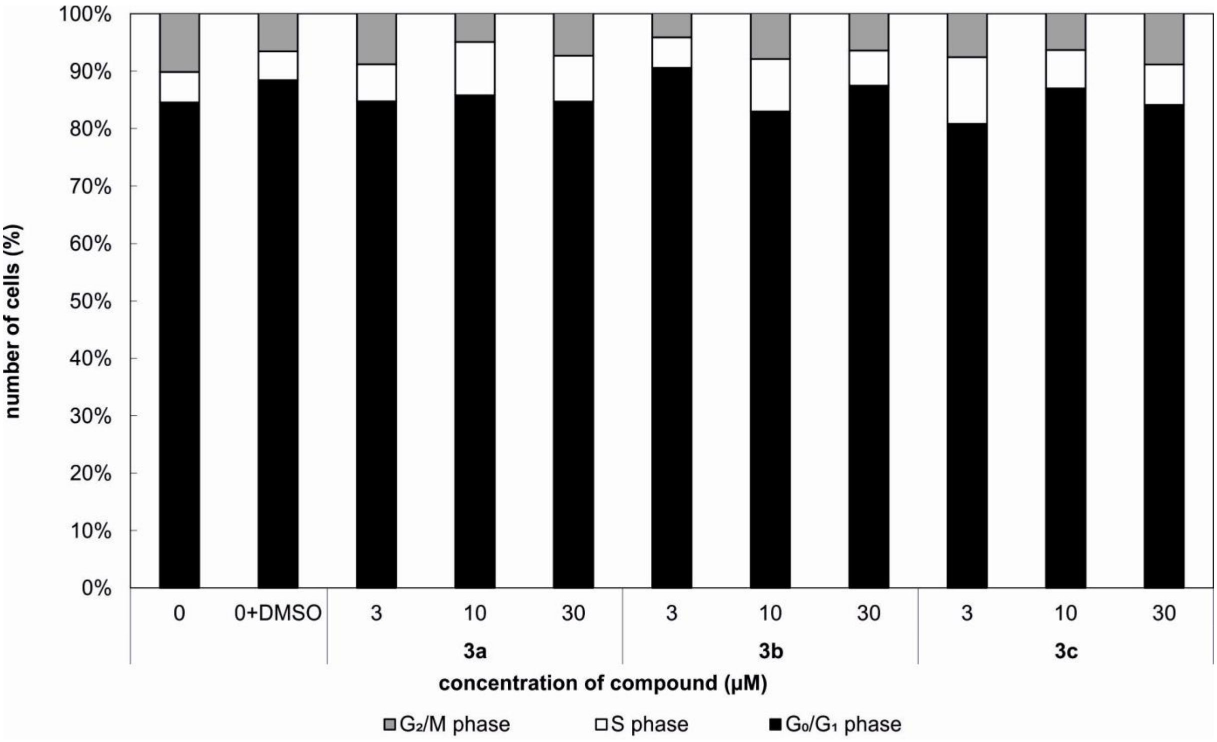

Figure S1B. Apoptosis of G-361 cancer cells

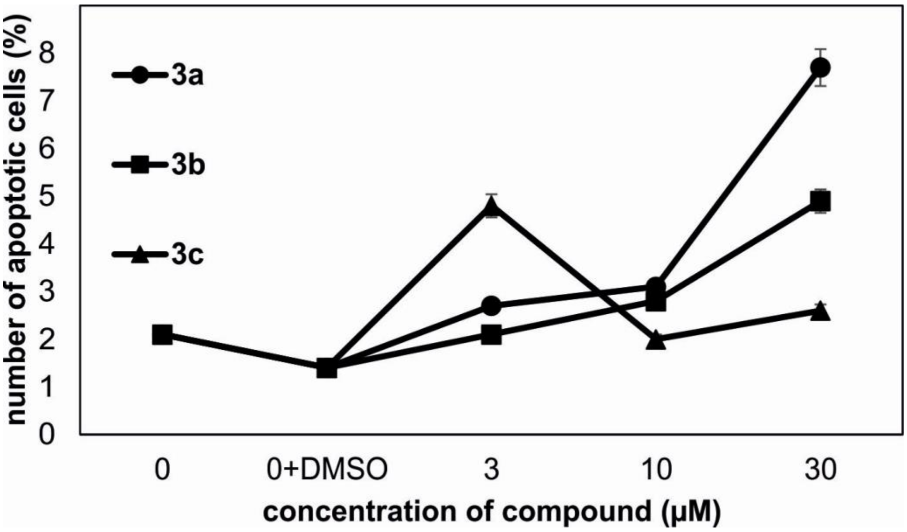

Figure S2. The level of protein markers of apoptosis (PARP, caspase-7 zymogen and fragment, Mcl-1, Bcl-2) detected by western blotting. β-Actin was used as loading control. G-361 cells were treated for 24 h by **3a**–**3c** (3, 10 and 30 µM). The bars 0 are control untreated cells, the bars 0+ are control cells with DMSO. Presented results presented are from a representative experiment repeated three times.

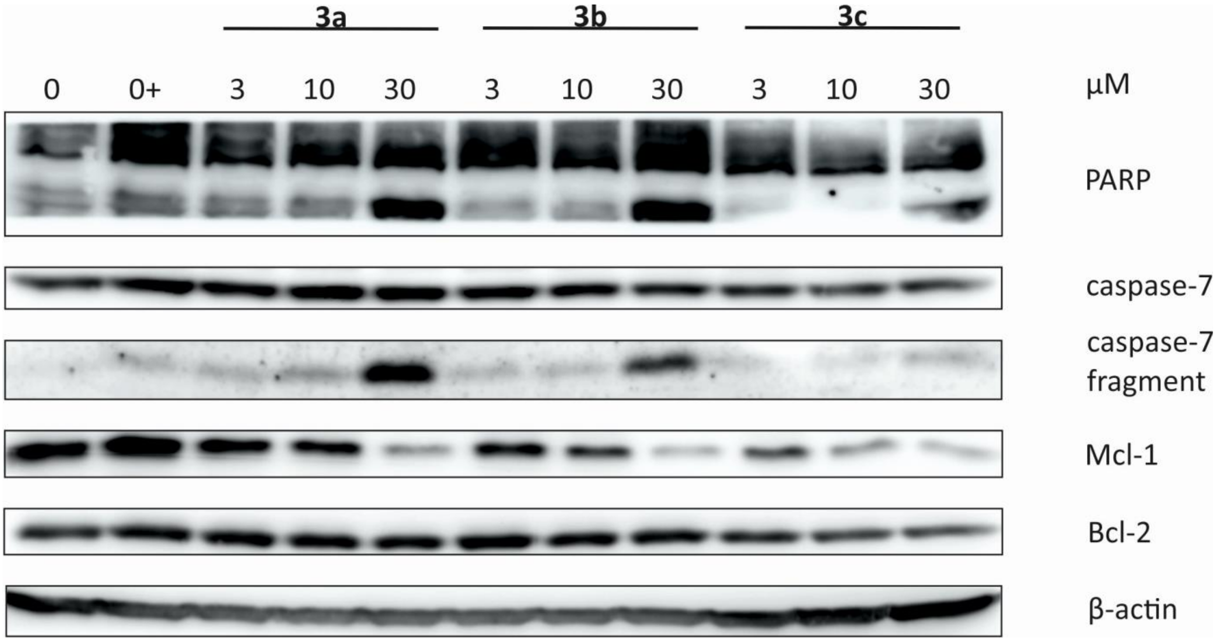

Figure S3. Activity of caspase-3/7 after 24 h treatment with **3a**–**3c** (3, 10 and 30 μM) in G-361 cells. The bars 0 are control untreated cells, the bars 0+ are control cells with DMSO. The experiment was repeated at least twice in duplicates.

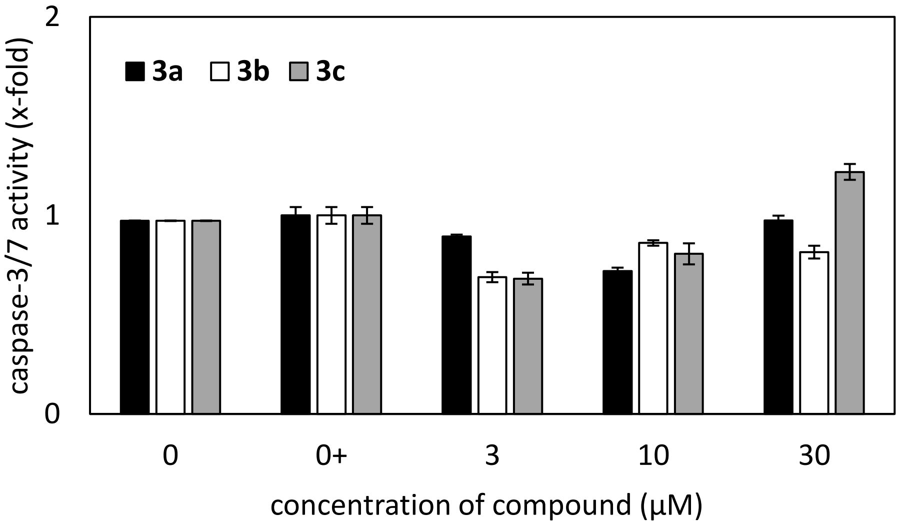

4. *In silico* calculated physico-chemical and ADME parameters.

In addition to what was written in the main text of this paper, **3a**–**4c** show only parameters log *PB* and log *BB* within the recommended range for those parameters. Those two parameters are connected with the activity of the compound on central nervous system (CNS) and with a potential ability of the compound to penetrate through the blood-brain barrier (log *PS* and log *PS* * $f_{u,brain}$) [44]. All those values calculated for **3a**–**4c** appear as negative values, and these compounds show no CNS activity, i.e., they cannot be considered as adaptogens (**Table 2**). It seems that calculation of physico-chemical and ADME parameters for **3a**–**4c** resulted in no substantial support for the experimental data that was observed in our recent papers to a larger extent. Our hypothesis is that the reason may consist in their supramolecular characteristics that are not involved in this type of calculation. **Figures S4** show the calculated positions of oleanolic acid (**1**), tryptamine, **3a** and **4a** (green dots) in comparison with those of CNS active drugs (blue dots) and CNS inactive drugs (orange dots) tested for the CNS activity.

Figure S4A. Oleanolic acid (**1**; position given by the green dot)

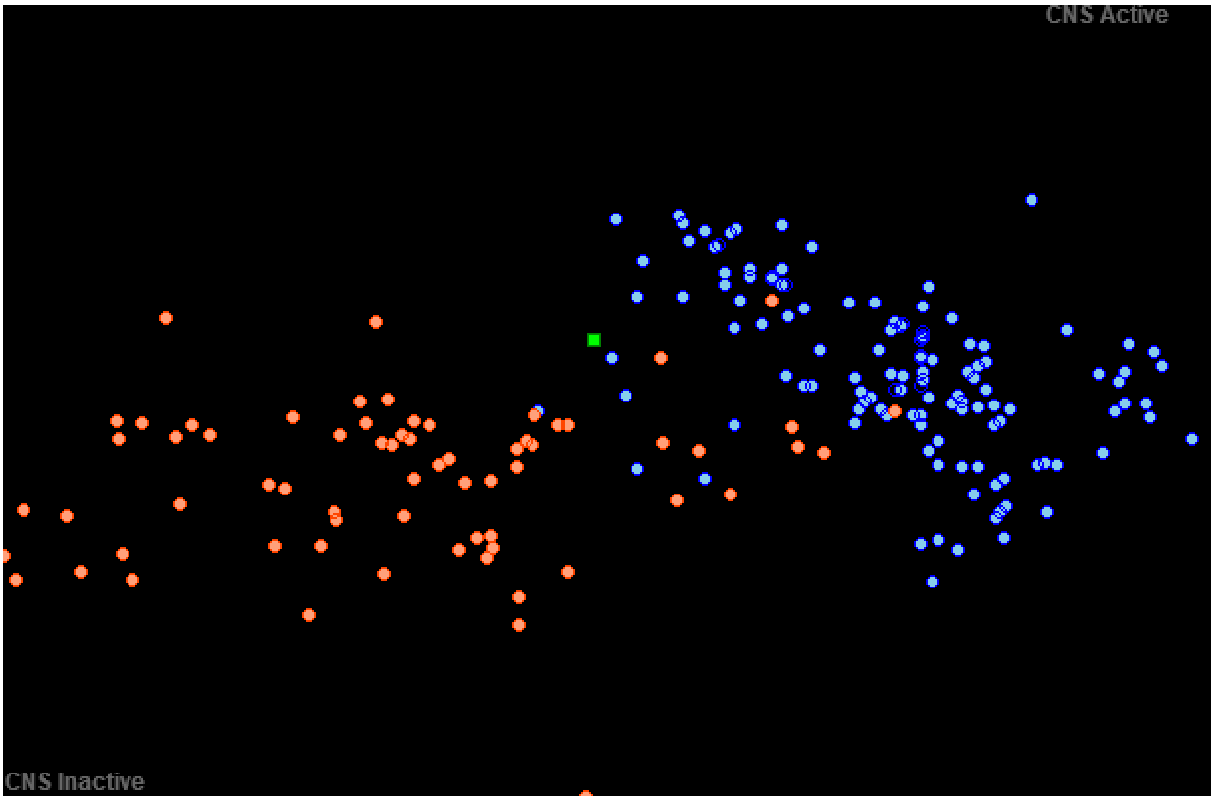

Figure S4B. Tryptamine (position given by the green dot)

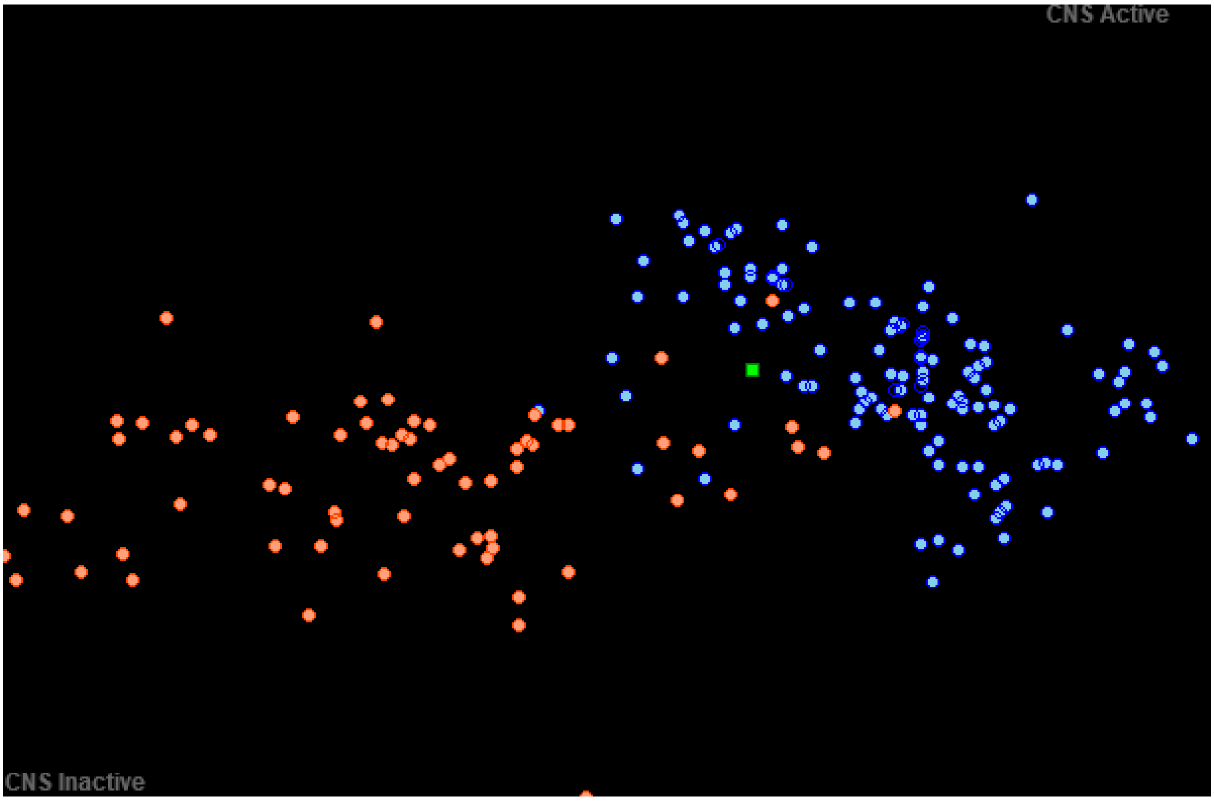

Figure S4C. Compound **3a** (green dot is on the left border in the figure)

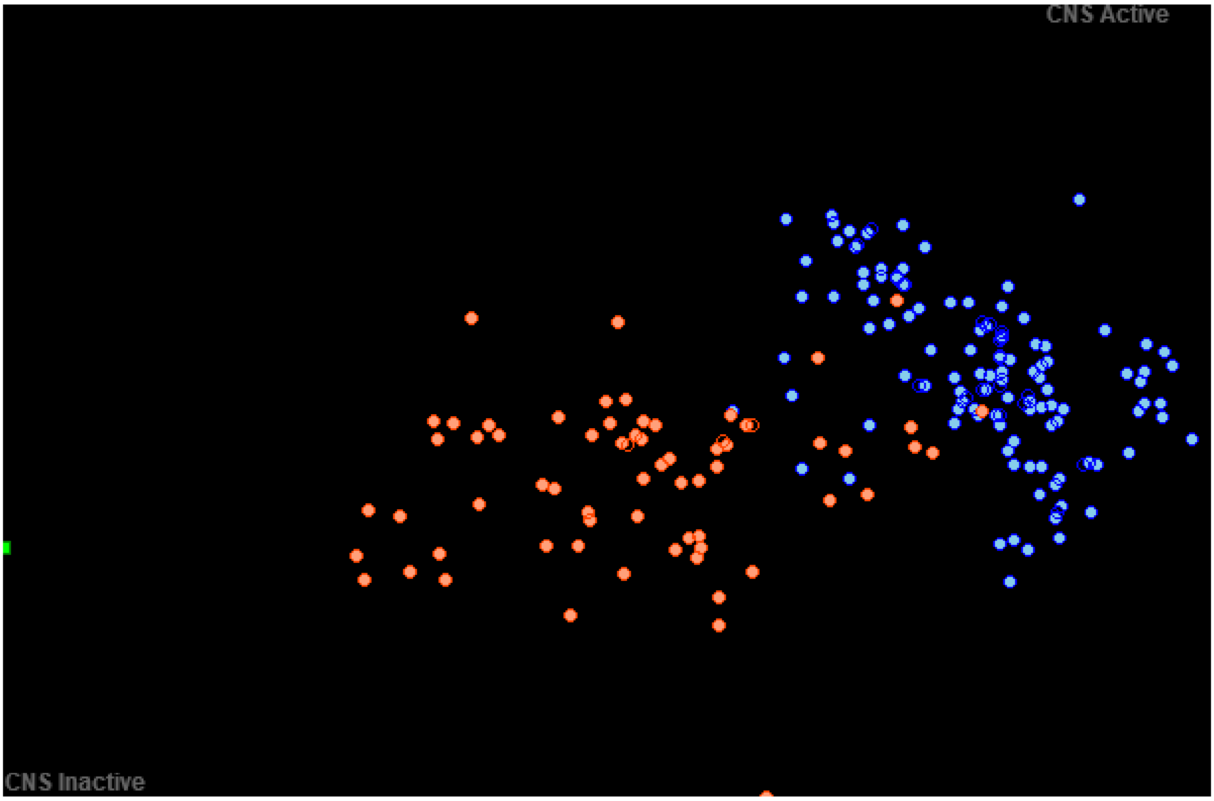

Figure S4D. Compound **4a** (green dot is on the left border in the figure)

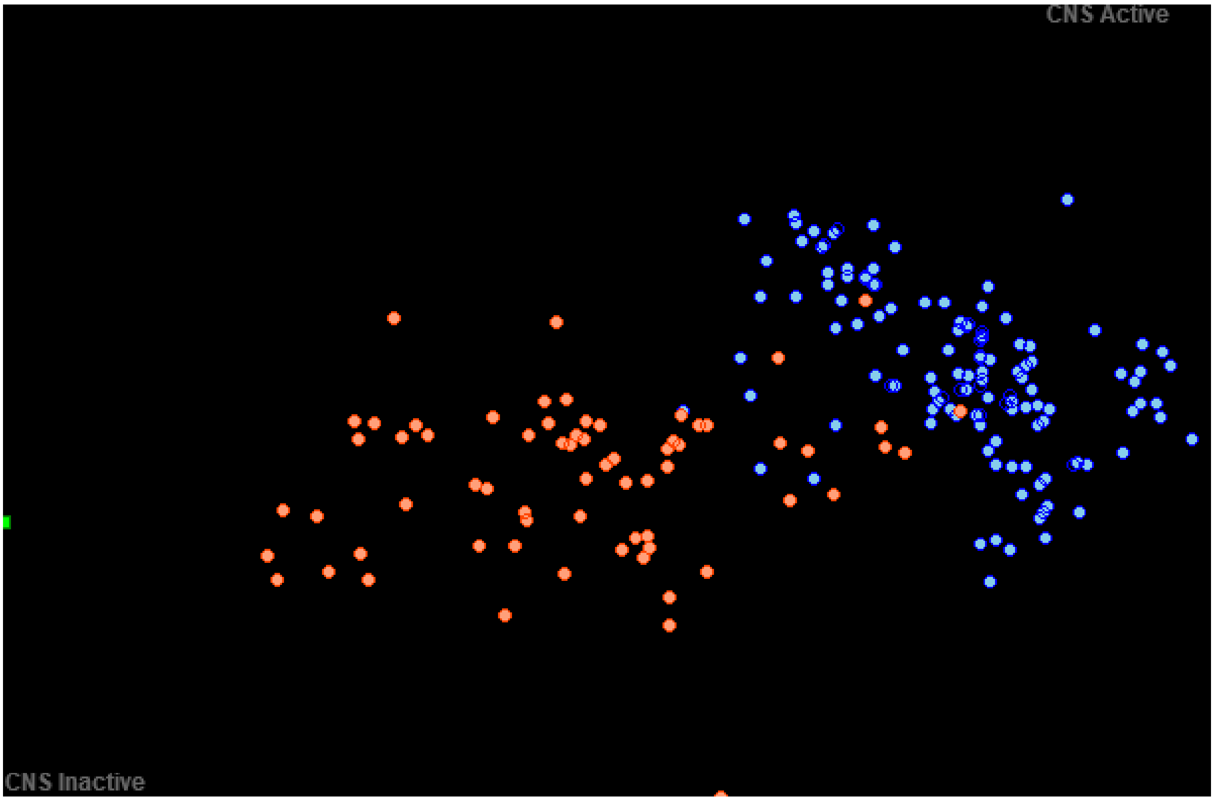

5. Investigation of supramolecular self-assembly by UV spectroscopy

Figures S5 show several example UV spectra measured with **3a**–**3c** in the methanol/water systems. **Figure S5A** shows both, irregularity and decrease in the absorbance values measured in the methanol/water systems with a ratio of the solvents methanol/water (60/40 and 40/60) at the constant concentration of **3a**. **Figure S5A** shows that in the methanol/water nixture (40/60) almost immediate formation of a supramolecular system appears, because the UV spectrum lost its characteristic maxima describing **3a**. The same course of the self-assembly can be seen with **3b** (**Figure S5C**) and **3c** (**Figure S5E**) as well. However, if the self-assembly of **3a**–**3c** is studied in different time intervals since the beginning of the measurement (hour 1, 4, 24 and 96), the supramolecular characteristics of the studied compounds are different.

Compound **3a**: Almost immediate formation of a supramolecular system was observed in the solvent mixtures containing 40 %, 20% and 0 % of methanol, and slow formation of self-aggregation within 24 h since the beginning was observed with 60 % of methanol in the solvent mixture. No self-assembly was found in 100 % and 80 % methanol (**Figure S5B**).

Compound **3b**: Using **3b**, irregularities were pronounced in the most remarkable way (**Figure S5D**). The absorbance maximum decreased in the solvent mixtures containing 60 % of methanol within 24 h. An almost immediate decrease of the absorbance maximum appeared in the solvent mixtures containing 40 % of methanol. However, with 20 % and 0 % of methanol, a slow decrease of both absorbance maxima appeared together with the maximum shift towards higher wavelength. No detectable self-assembly was found in 100 % and 80 % methanol.

Compound **3c**: Using **3c**, irregularities due to self-assembly were less visible than with **3b** (**Figure S5F**). Almost immediate disappearing of the absorbance maxima with 40 %, 20 % and 0 % methanol in the solvent mixture was accompanied by less apparent shifting of the

absorbance maxima towards higher wavelength. A slow formation of self-aggregation within 24 h since the beginning was again observed with 60 % of methanol in the solvent mixture. No self-assembly was found in 100 % and 80 % methanol.

Figure S5A. UV spectra of **3a** in the methanol/water (60 / 40) and (40 / 60) systems.

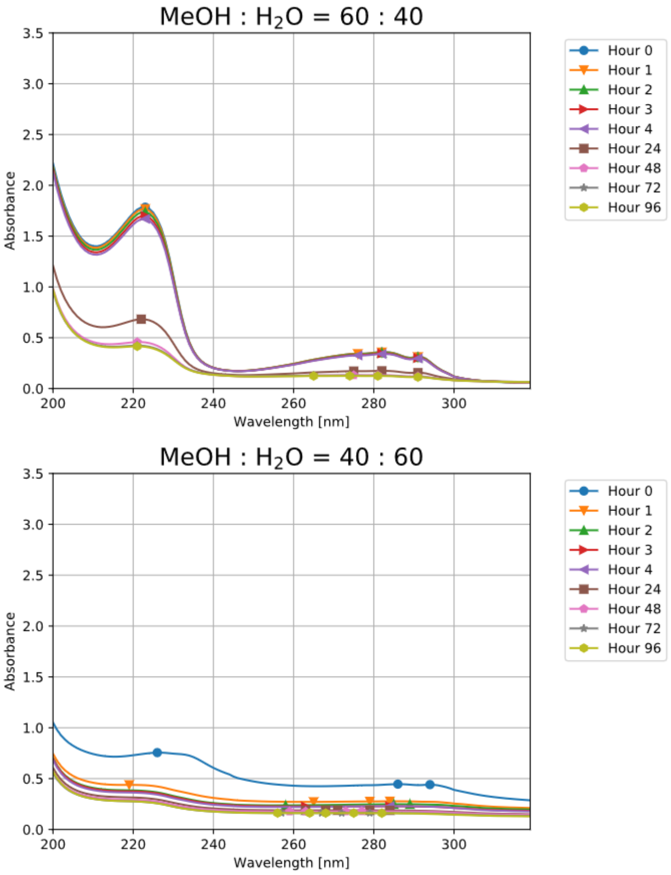

Figure S5B. UV spectra of **3a** in different methanol/water systems in hour 1, 4, 24 and 96.

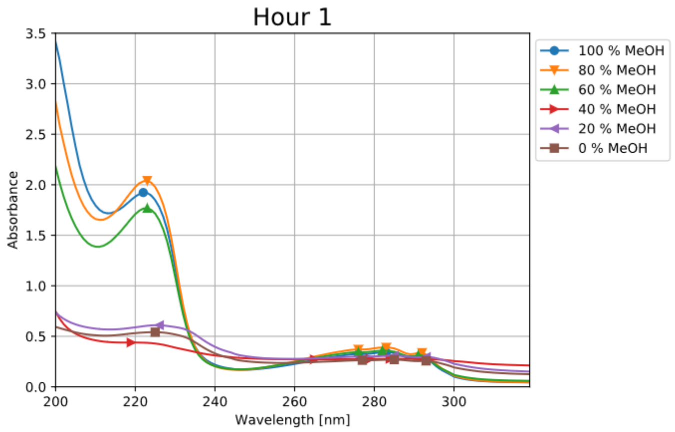

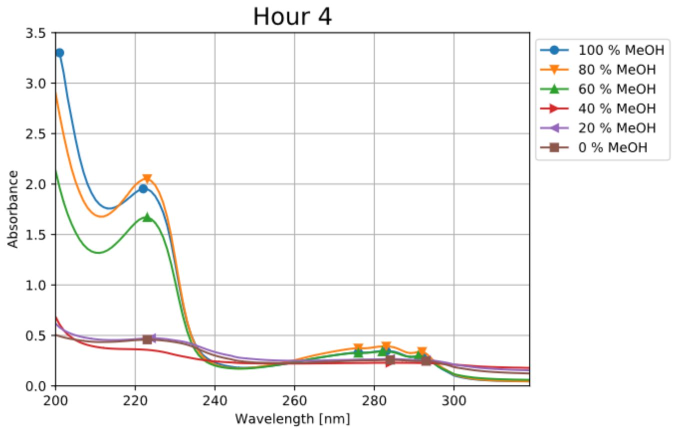

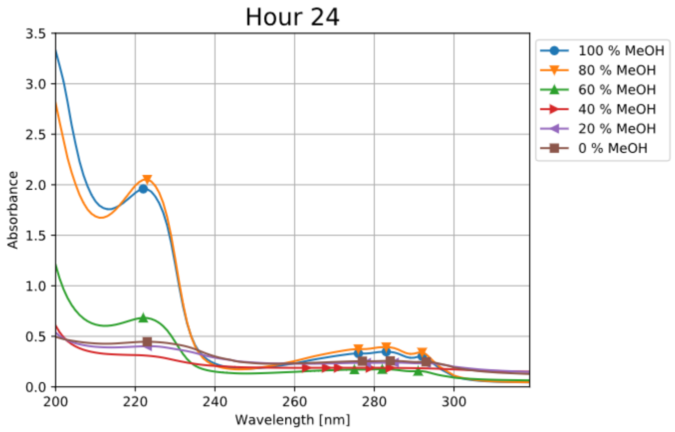
Hour 24
Absorbance
Wavelength [nm]
100 % MeOH
80 % MeOH
60 % MeOH
40 % MeOH
20 % MeOH
0 % MeOH

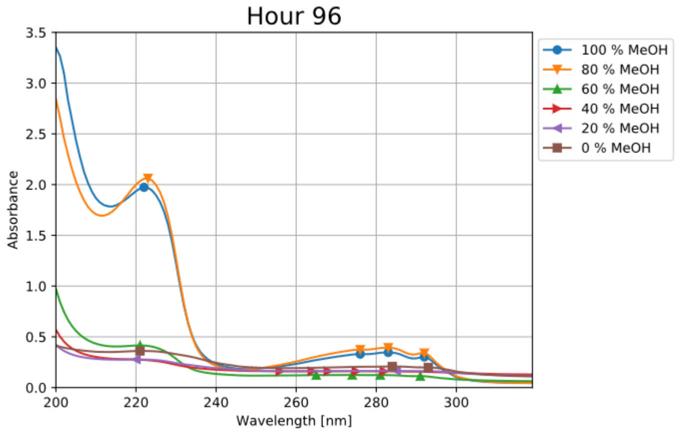
Hour 96
Absorbance
Wavelength [nm]
100 % MeOH
80 % MeOH
60 % MeOH
40 % MeOH
20 % MeOH
0 % MeOH

Figure S5C. UV spectra of **3b** in the methanol/water (60 / 40) and (40 / 60) systems.

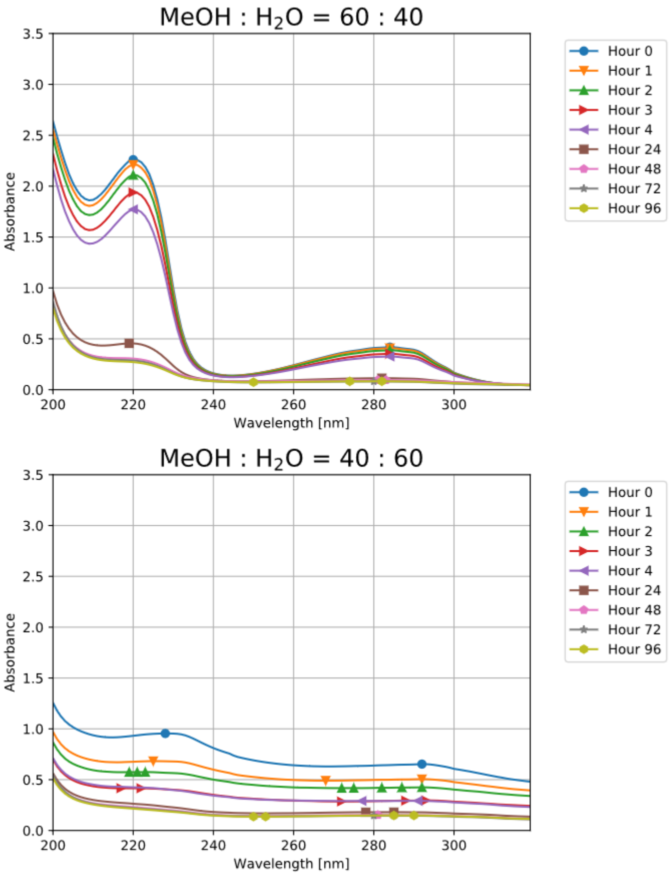

Figure S5D. UV spectra of **3b** in different methanol / water systems in hour 1, 4, 24 and 96.

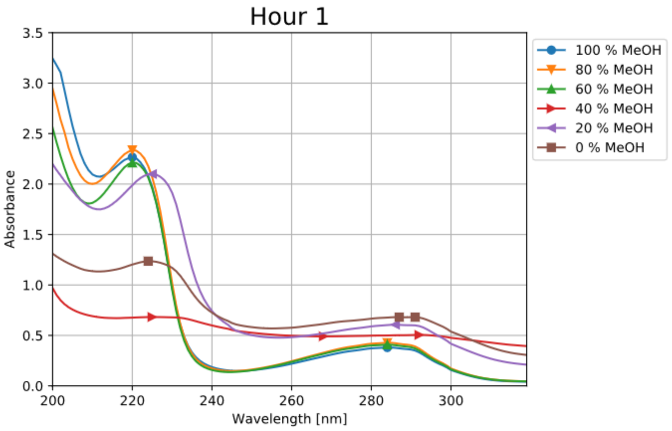

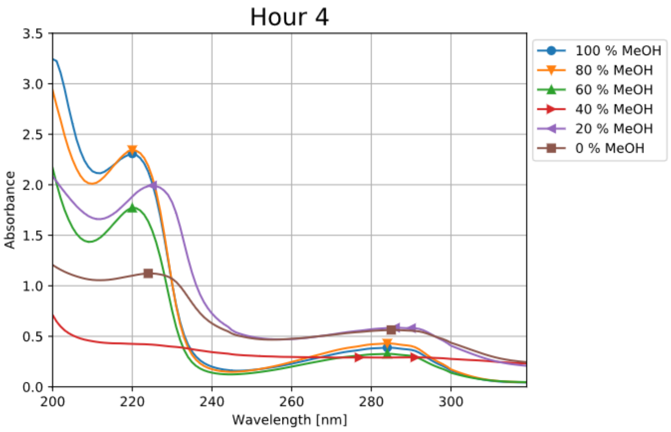

Hour 4
Absorbance
Wavelength [nm]
100 % MeOH
80 % MeOH
60 % MeOH
40 % MeOH
20 % MeOH
0 % MeOH

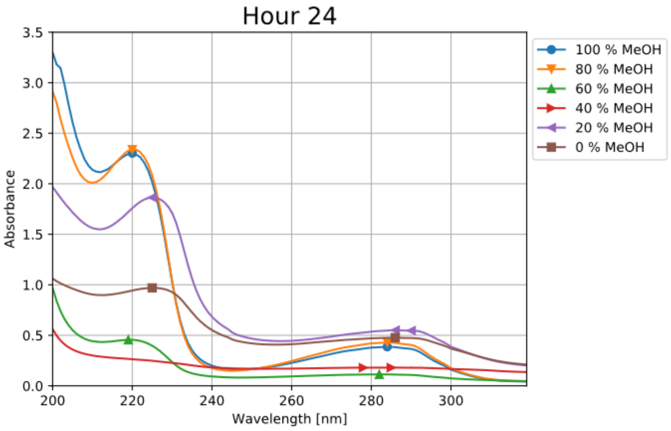

Hour 24
Absorbance
Wavelength [nm]
100 % MeOH
80 % MeOH
60 % MeOH
40 % MeOH
20 % MeOH
0 % MeOH

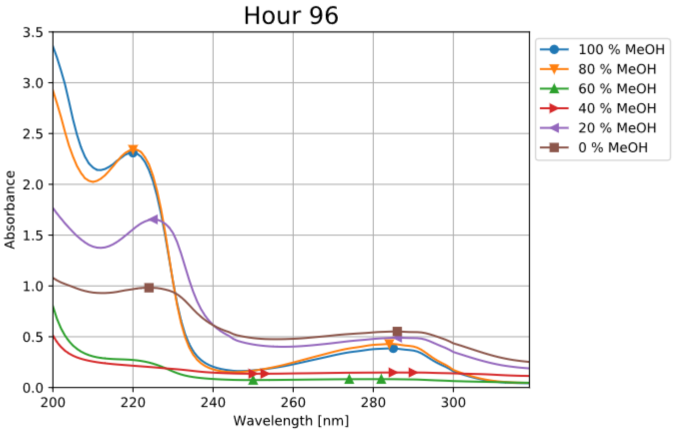

Hour 96
Absorbance
Wavelength [nm]
100 % MeOH
80 % MeOH
60 % MeOH
40 % MeOH
20 % MeOH
0 % MeOH

Figure S5E. UV spectra of **3c** in the methanol/water (60 / 40) and (40 / 60) systems.

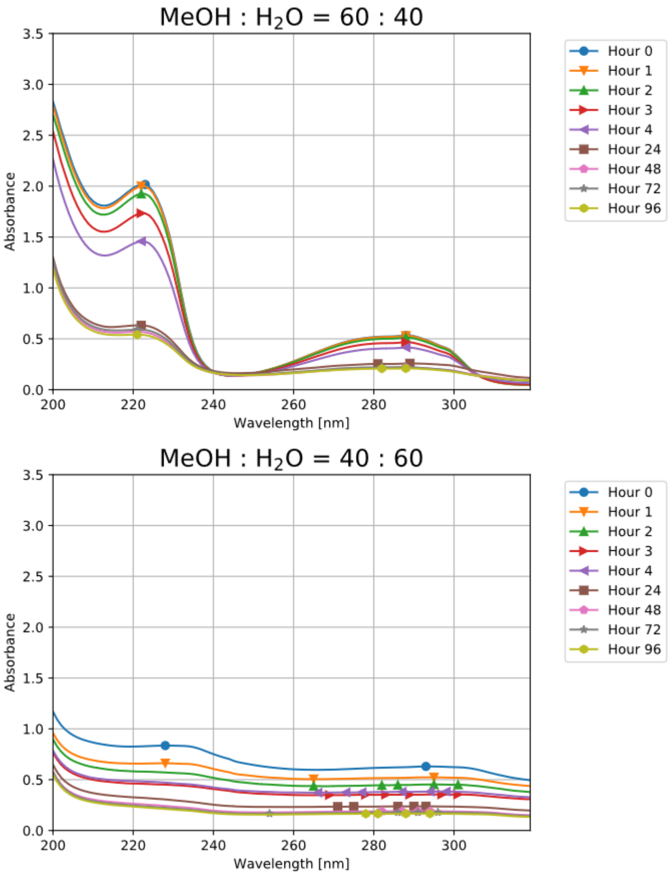

Figure S5F. UV spectra of **3c** in different methanol / water systems in hour 1, 4, 24 and 96.

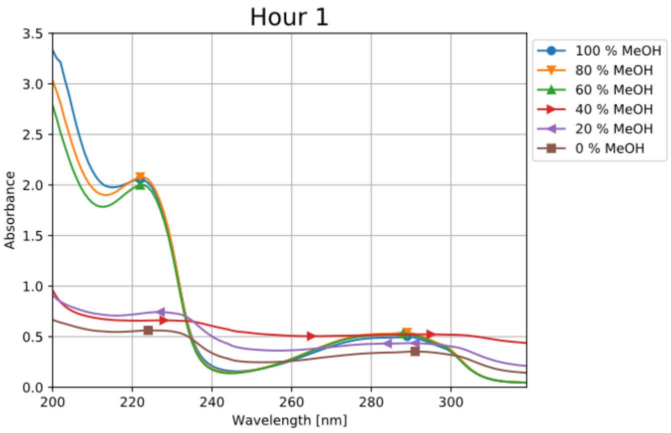

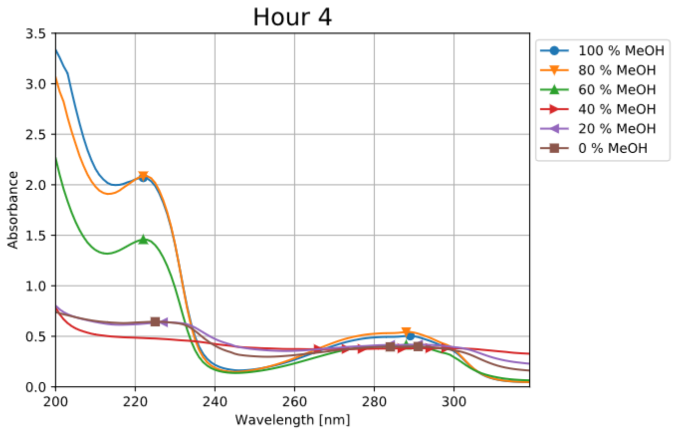

Hour 4
Absorbance
Wavelength [nm]
100 % MeOH
80 % MeOH
60 % MeOH
40 % MeOH
20 % MeOH
0 % MeOH

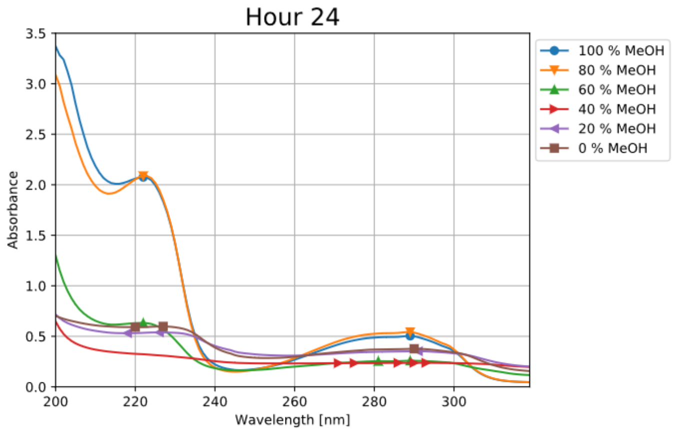

Hour 24
Absorbance
Wavelength [nm]
100 % MeOH
80 % MeOH
60 % MeOH
40 % MeOH
20 % MeOH
0 % MeOH

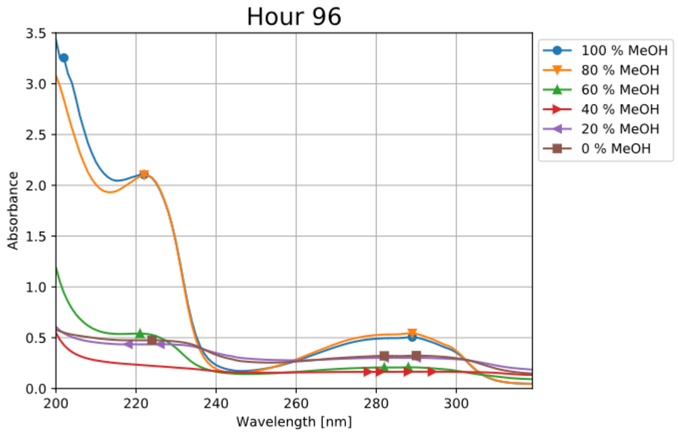

Hour 96
Absorbance
Wavelength [nm]
100 % MeOH
80 % MeOH
60 % MeOH
40 % MeOH
20 % MeOH
0 % MeOH

Graph S1. Blotted membranes with proteins colored by Ponceau-S (3-hydroxy-4-(2-sulfo-4-[4-sulfophenylazo]phenylazo)-2,7-naphthalenedisulfonic acid sodium salt).

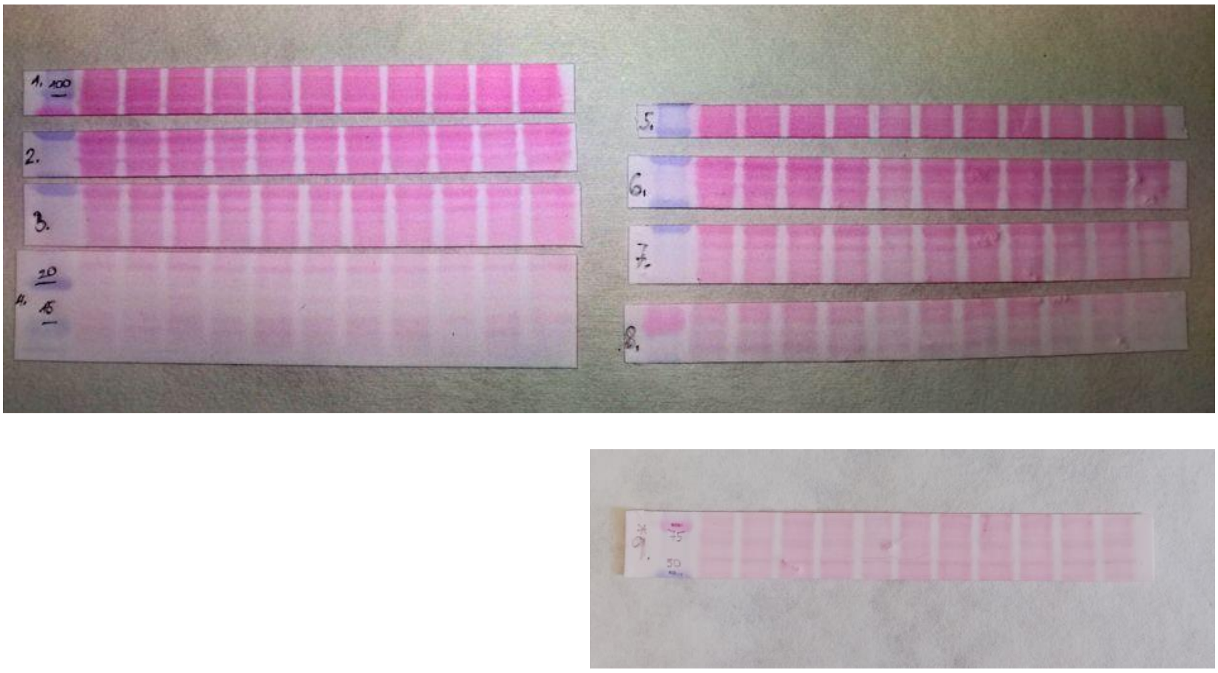

Supplement: Supplementary file 1 [file plants-10-02082-s001.zip › plants-1380129-supplementary.pdf]
